# Supplementary material for: Reference genome assemblies reveal the origin and evolution of allohexaploid oat
Source: Nat Genet. 2022 Jul 18;54(8):1248–58. doi: 10.1038/s41588-022-01127-7 (PMC9355876; doi:10.1038/s41588-022-01127-7)
Supplement: Supplementary file 1 — Supplementary Notes [file 41588_2022_1127_MOESM1_ESM.pdf]

nature portfolio

https://doi.org/10.1038/s41588-022-01127-7

Supplementary information

Reference genome assemblies reveal the origin and evolution of allohexaploid oat

In the format provided by the authors and unedited

Supplementary Notes

Contents

1. Genome sequencing and assembly 4

1.1 Plant material 4

1.2 Short-read sequencing 4

1.3 Nanopore sequencing 5

1.4 Hi-C library preparation and sequencing 6

1.5 PacBio Iso-Seq 7

1.6 Contig assembly, polishing, and evaluation 8

1.7 Chromosome construction 10

1.8 Quality Assessment 12

1.9 Identification of centromeric regions 13

2. Genome annotation 15

2.1 Protein-coding gene annotation 15

2.2 Functional annotation of gene models 17

2.3 Noncoding RNA prediction 17

2.4 Repetitive element annotation 17

2.5 Pseudogene annotation 18

3. Subgenome assignment, validation, and nomenclature 19

4. Phylogenomics and comparative genomic analyses of cereal crops 22

4.1 Phylogenetic tree construction and divergence time estimation 22

4.2 Gene family analysis 23

4.3 Karyotype evolution 24

5. The evolution and allopolyploidization history of oat 25

5.1 Whole-genome sequencing-based analyses 25

5.1.1 Plant material 25

5.1.2 Whole-genome sequencing 25

5.1.3 Identity plots 25

5.1.4 Variant calling 26
5.1.5 Phylogenetic tree construction using SNPs 27
5.2 Transcriptome sequencing-based analyses 27
5.2.1 Plant growth and RNA isolation and sequencing 27
5.2.2 Transcript assembly and CDS prediction 28
5.2.3 Phylogenetic tree construction and divergence time estimation 28
5.3 Organelle-based analyses 29
6. Subgenome evolution 30
6.1 Chromosome rearrangement 30
6.1.1 Synteny analysis 30
6.1.2 Fluorescence in situ hybridization (FISH) 31
6.1.3 Functional enrichment of genes involved in translocations 33
6.2 Subgenome contents 34
6.2.1 k-mer distribution 34
6.2.2 Genome size variation analyses 34
6.2.3 Repetitive DNA comparison 35
6.2.4 Gene content comparison 35
6.2.5 Gene loss and retention 36
6.2.6 *Ka/Ks* analysis 37
6.3 Subgenome dominance 38
6.3.1 Plant materials and transcriptome sequencing 38
6.3.2 Quantification of gene expression levels 38
6.3.3 Analysis of homoeologous gene expression 39
6.3.4 Relationship between gene expression and TE-density 39
7. Genes related to agronomic traits 41
7.1 Identification of R-genes 41
7.2 Identification of candidate genes for the hulless trait 42
7.2.1 Plant materials 42

7.2.2 DNA isolation, library construction, and DNA sequencing..................42
7.2.3 Sequence data analysis and SNP calling..43
7.2.4 Genome-wide association analysis 44
7.2.5 Transcriptome comparison between hulled and hulless oats 44
7.2.6 Sequence comparison of gene *A.satnudSFS4D01G000045* 45
7.2.7 Kompetitive Allele-Specific PCR (KASP) validation 45

Supplementary Notes

1. Genome sequencing and assembly

1.1 Plant material

The hulless hexaploid oat (*Avena sativa* L. ssp. *nuda*, 2n=6x=42, AACCDD) landrace cv. 'Sanfensan', the diploid species *A. longiglumis* (accession CN 58139, 2n=2x=14, AlAl), and the tetraploid species *A. insularis* (accession CN 108634, 2n=4x=28, CCDD) were chosen for whole-genome sequencing. 'Sanfensan' is a traditional hulless variety that has a long cultivation history in Shanxi, China, which is thought to be the geographical region where hulless oat originated. *A. longiglumis* and *A. insularis* are thought to be the extant diploid and tetraploid species most closely related to hexaploid oat[1] (**Supplementary Table 1**).

1.2 Short-read sequencing

High-quality genomic DNA was isolated from fresh leaf tissue using the Qiagen DNeasy Plant Mini Kit. Two sequencing platforms, the Illumina HiSeq Xten (Illumina, USA) and the MGISEQ-2000 (BGI, China), were used for genome sequencing. Illumina sequencing libraries were prepared using the TruSeq Nano DNA HT Sample preparation kit (Illumina, USA) following the manufacturer's recommendations. MGI libraries were constructed as follows: briefly, 1-1.5 μg of genomic DNA was randomly fragmented with a Covaris instrument. DNA fragments between 200-400 bp in length were selected using an Agencourt AMPure XP-Medium kit, followed by end repair, 3' adenylation, and adapter ligation. After PCR enrichment, the PCR products were recovered using the AxyPrep Mag PCR Clean-Up kit. The double-stranded PCR products were heat denatured and

circularized using the splint oligo sequence. Single-stranded circular DNA (ssCir DNA) was formatted as the final library and qualified according to QC procedures. The qualified libraries were sequenced on the Illumina HiSeq X-Ten or MGISEQ-2000 platform at the Genome Center of GrandOmics (Wuhan, China) (**Supplementary Table 1**).

1.3 Nanopore sequencing

The Oxford Nanopore Technologies (ONT) system was used to sequence all three oat genomes in this study. The ONT ultralong strategy was selected for the whole genome sequencing of the hexaploid species because of its large, complex genome. For this purpose, approximately 8-10 μg of genomic DNA was size-selected (>50 kb) with the SageHLS HMW library system (Sage Science, USA) and processed using the Ligation sequencing 1D kit (SQK-LSK109, Oxford Nanopore Technologies, UK) according to the manufacturer's instructions. For the diploid and tetraploid samples, ONT regular sequencing was used for whole-genome sequencing. A total of 3-4 μg DNA per sample was used as input material for the ONT library preparation. After a sample was qualified, size-selection of long DNA fragments was performed using the PippinHT system (Sage Science, USA). The ends of the DNA fragments were repaired and single dA residues were added to the 3' ends with the NEBNext Ultra II End Repair/dA-tailing Kit (Catalog number E7546). The adapter provided in the SQK-LSK109 kit (Oxford Nanopore Technologies, UK) was used for the subsequent ligation reaction, and the DNA library was quantified using a Qubit® 4.0 Fluorometer (Invitrogen, USA). DNA libraries with approximately 800 ng and 700 ng of inserts were constructed for ONT ultralong and ONT regular sequencing, respectively, and were sequenced on the PromethION platform (Oxford Nanopore Technologies, UK)

at the Genome Center of Grandomics (Wuhan, China).

Base calling was performed with the ONT basecaller Guppy (v3.2.2) with the following parameters: -c dna_r9.4.1_450bps_fast.cfg. The raw Nanopore reads were filtered, and only reads with a mean_qscore_template ≥7 was retained for downstream analyses. A total of 71, 7, and 8 libraries were sequenced for ‘Sanfensan’, *A. insularis*, and *A. longiglumis*, generating 1,260.30 Gb, 481.39 Gb, and 268.74 Gb of raw data, respectively. The post-filtered Nanopore reads produced a total of 1,027.83 Gb, 374.77 Gb, and 218.67 Gb of sequencing data, providing approximately 100, 60 and 60× coverage of the genomes, respectively. The ONT ultralong sequencing produced much longer reads than did the ONT regular sequencing, as reflected by the longer maximum read length, average read length, and read N50. The detailed information on the data generated from each ONT cell is summarized in **Supplementary Table 2**.

1.4 Hi-C library preparation and sequencing

The Hi-C libraries were prepared as described previously[2] with some modifications. In brief, oat plants (*A. sativa* ssp. *nuda* cv. ‘Sanfensan’ and *A. insularis*) were grown in a growth chamber for two weeks. Samples of 2-4 g of tender young seedling leaves were harvested, cut into pieces of ca. 2 cm^2, and transferred to 50 ml tubes containing 15 ml of ice-cold nuclear isolation buffer (NBE) with 2% formaldehyde, followed by vacuum infiltration (400 mbar), and incubation with a supplemented crosslinking agent for 1 h. Crosslinking was quenched by adding 2 M glycine to a final concentration of 0.125 M with incubation for 5 min under vacuum, followed by fixation on ice. The fixed leaf pieces were then washed three times with sterile Milli-Q water, ground in liquid nitrogen, and used for nucleus isolation. The isolated nuclei were purified, checked for quality and quantity and digested with 100 units of

the restriction endonuclease *DpnII*. The next steps were Hi-C-specific, including marking the DNA ends with biotin-14-dATP and performing blunt-end ligation of the crosslinked fragments. After ligation, crosslinking was reversed by overnight incubation with proteinase K at 65 °C. Biotin-14-dATP was further removed from non-ligated DNA ends using the exonuclease activity of T4 DNA polymerase. DNA was purified by phenol:chloroform (1:1) extraction, precipitated, and washed as described by previous study[2]. The purified DNA was physically sheared to a size of 300-600 bp by sonication and was size-fractionated using standard 2% agarose gel electrophoresis to obtain fragments in the range of 300-600 bp. The ends of the DNA fragments were blunt-end repaired, dA-tailed, and subjected to Illumina PE sequencing adapter addition, followed by purification through biotin-streptavidin-mediated pulldown. PCR amplification was conducted with 12-15 cycles of PCR to enrich the ligation products. After a quality check, the Hi-C libraries were sequenced on Illumina HiSeq X-Ten instruments to generate 150-base paired-end reads. A total of 1,312.83 Gb and 816.93 Gb of raw Hi-C sequencing data was generated for ‘Sanfensan’ and *A. insularis*, respectively (**Supplementary Table 1**).

1.5 PacBio Iso-Seq

The three ONT-sequenced oat species were grown in the greenhouse or the field to different growth stages, and the following seven types of samples were collected for RNA isolation: two-week-old seedlings, flag leaves at the booting (Zadok’s 45) and heading (Zadok’s 58) stages, and panicles at the booting (Zadok’s 45), heading (Zadok’s 50 and 58), and grain dough (Zadok’s 83) stages. The RNA extracted from the seven types of samples was mixed in equal amounts and subjected to quality

checks using 0.75% agarose gel electrophoresis, a Qubit fluorometer (Thermo Fisher), and an Agilent 2100 BioAnalyzer. Full-length cDNA Iso-Seq template libraries were prepared by following the protocol provided by Pacific Biosciences with some modifications. For each sample, 500 ng of total RNA was subjected to reverse transcription using a SMARTer PCR cDNA Synthesis Kit (Clontech). Large-scale PCR was then performed to amplify the cDNAs using KAPA HiFi PCR Kits. To minimize artefacts during large-scale amplification, the number of cycles was optimized and determined to be 14. After large-scale PCR amplification, the resulting PCR products were purified using 1× AMPure PB Beads, followed by additional purification with 0.4× AMPure PB Beads. The purified amplicons were fractionated, and fractions with sizes between 0.5-6 kb were harvested using the BluePippin™ Size Selection System to generate SMRTbell™ libraries using the PacBio Template Prep Kit. The SMRTbell templates were then sequenced on a PacBio Sequel II machine at the Genome Centre of Grandomics (Wuhan, China). A total of 46,759,952, 26,389,556, and 13,550,480 reads covering 81.14 Gb, 49.94 Gb, and 25.74 Gb were generated for the hexaploid, tetraploid, and diploid oat species, respectively (**Supplementary Table 1**).

1.6 Contig assembly, polishing, and evaluation

To provide guidance regarding genome assembly, the genome sizes of the three *Avena* species were estimated by counting the 17-mer frequencies among the clean short reads with Jellyfish (v2.0)[3] software, which resulted in estimated genome sizes of 10.98 Gb, 7.96 Gb, and 4.04 Gb for ‘Sanfensan’, *A. insularis*, and *A. longiglumis*, respectively.

De novo genome assembly was performed based on Nanopore long reads using the

NextDenovo (v2.0-beta.1) pipeline (https://github.com/Nextomics/NextDenovo). NextDenovo is a string graph-based de novo assembler for long reads. It uses a “correct-then-assemble” strategy similar to Canu, but requires significantly less computing resources and storage. The authors benchmarked NextDenovo against six other assemblers using ONT long reads from human and *Drosophila melanogaster* DNA, and PacBio long reads from *Arabidopsis thaliana*. The results showed that NextDenovo generates more contiguous assemblies with fewer contigs compared to the other tools without compromising the assembly accuracy in terms of assembly consistency and single-base accuracy (https://nextdenovo.readthedocs.io/en/latest/index.html). NextDenovo has been used for genome assembly in various species with different genome sizes and heterozygosity rates including African lungfish[4], elephant grass[5], and the moth *Ephestia elutella*[6]. To this end, cleaned Nanopore reads were first self-corrected using the NextCorrect module with the default settings, and the corrected reads were then assembled into contigs to obtain the draft assembly using NextDenovo (parameters: reads_cutoff: 1k and seed_cutoff: 54 k for ‘Sanfensan’, 25 k for *A. insularis* and *A. longiglumis*). The sizes, contig numbers and contig N50 values of the draft genome assemblies are summarized in **Supplementary Table 3.**

To obtain a high-quality genome assembly, the draft assemblies were further improved by using short reads and corrected Nanopore long reads. For this purpose, raw Illumina or MGI reads were processed with Trimmomatic (v.0.40)[7] to remove adapter sequences, low-quality reads, and short reads <70 bp in length. This produced 649.68 Gb, 404.97 Gb, and 204.68 Gb of clean reads for ‘Sanfensan’, *A. insularis*, and *A. longiglumis*, respectively, achieving ~50× coverage of all three genomes. Two steps were included to improve the draft genome assemblies; first, using mininmap2

(v2.18)[8] (parameters: -x map-ont) and Racon (v1.4.21)[9] (the default settings), the corrected Nanopore reads were aligned to the draft assembly for correction, and second, the filtered short reads were employed to polish the draft assemblies using NextPolish (v1.0.5). After three rounds of Racon polishing and four rounds of NextPolish polishing, the corrected genomes of 'Sanfensan', *A. insularis*, and *A. longiglumis* had sizes of 10,759,349,041 bp, 7,520,994,703 bp, and 3,738,867,912 bp, respectively, which accounted for 97.98%, 94.49%, and 92.54% of the genome sizes estimated from k-mer analysis (**Supplementary Table 3**).

Contigs with contaminants were removed by NCBI-blast (version 2.2.28+) against the NT database. To this end, contigs >1 Mb in length were split into 50 kb bins which were then used as queries to search the NT database. These analyses detected three, eight, and four contigs with contaminants in the 'Sanfensan', *A. insularis* and *A. longiglumis* genome assemblies, totaling 1,915,696 bp, 1,976,263 bp, and 2,319,367 bp, respectively. The average GC contents of the 'Sanfensan', *A. insularis*, and *A. longiglumis* genomes were 43.80%, 43.48%, and 44.35%, respectively, which are similar to those of most cereals including the diploid oats reported in a previous study[10]. The average coverage was between 40× and 60× for all three genome assemblies, and only one major peak was present in the contig distributions, suggesting few collapsed haplocontigs (i.e., contigs representing both haplotypes of the genome) in these genome assemblies (**Extended Data Fig. 2e**).

1.7 Chromosome construction

The genome assembly of the diploid species *A. longiglumis* was anchored and arranged into seven pseudomolecules with RaGOO (v1.1)[11] using the previously published reference genome of the *Avena* A-genome diploid *A. atlantica*[10] as the

reference. For the tetraploid and hexaploid assemblies, contig anchoring and orientation were performed with the aid of Hi-C data (**Extended Data Fig. 1**). For this purpose, the raw reads from the Hi-C libraries were filtered using fastp[12] with the default settings, resulting in a total of 803,368,743,610 bp and 1,296,125,167,024 bp of clean data. The clean Hi-C reads were then aligned to the assemblies using Bowtie2 (v.2.3.2)[13] with the end-to-end model (parameters: -very-sensitive -L 30), which resulted in 45.43% and 48.37% uniquely mapped paired-end reads out of the total ~2,691 million and ~4,221 million pairs of clean reads for *A. insularis* and 'Sanfensan', respectively. After considering the map positions and orientation of these unique reads, ~870 and ~1,372 million read pairs were retained as valid interaction pairs for *A. insularis* and 'Sanfensan', which represented 71.16% and 67.24% of the uniquely mapped reads, and 32.33% and 32.52% of the clean reads, respectively. Second, LACHESIS[14] software was used to cluster, order, and orient the contigs into chromosome-length pseudomolecules on the basis of the validated Hi-C dataset with the following parameters: CLUSTER MIN RE SITES=100; CLUSTER MAX LINK DENSITY=2.5; CLUSTER NONINFORMATIVE RATIO=1.4; ORDER MIN N RES IN TRUNK=60; ORDER MIN N RES IN SHREDS=60. The process contains an error-correction step, which broke 72 and 531 contigs from 'Sanfensan' and *A. insularis* into 182 and 1,339 contigs, respectively. Finally, 316 high-confidence 'Sanfensan' contigs were anchored to 21 pseudomolecules with a total length of 10,656,159,659 bp, accounting for 99.06% of the total assembly length, leaving 113 contigs comprising 101,273,686 bp unanchored, whereas 1,867 high-confidence *A. insularis* contigs were anchored to 14 pseudomolecules with a total length of 7,151,893,076 bp, accounting for 95.12% of the total assembly length.

1.8 Quality Assessment

We first calculated the NG(x) values for all integer thresholds (1-100%) and generated an NG graph for each of the three assembled genomes to estimate the continuity of the assemblies. We then mapped the Illumina paired-end data back to the assembled scaffolds using BWA (v0.7.10-r789)[15] with the default settings to assess the accuracy of the assembled genomes. We found that 99.75%, 99.69%, and 99.77% of the reads could be map onto the final assemblies of ‘Sanfensan’, *A. insularis*, and *A. longiglumis*, respectively, and 98.22%, 95.85%, and 94.12% were properly paired alignments. Alignments were sorted with SAMtools (v1.9)[16]. Variants were called using the HaplotypeCaller module of GATK (v4.1.9.0)[17] using the default parameters. We identified 98,885, 146,513, and 101,294 homozygous SNPs, as well as 19,444, 34,835, and 24,984 homozygous InDels in the ‘Sanfensan’, *A. insularis*, and *A. longiglumis* genomes, respectively, giving estimated overall nucleotide accuracy rates of 99.999%, 99.998%, and 99.997% for the three genomes. We also found 1,093,198, 1,851,651, and 1,408,256 heterozygous SNPs, as well as 66,019, 64,623, and 60,259 heterozygous InDels in the ‘Sanfensan’, *A. insularis* and *A. longiglumis* genomes, respectively, giving estimated overall heterozygosity rates of 0.011%, 0.025%, and 0.039% for these genomes, indicating that these species are largely homozygous. We used BUSCO (v5.2.2)[18] to examine genes conserved with the Embryophyta_odb10.2019-11-20 database. A total of 1,605 (99.44%), 1,603 (99.32%), and 1,590 (98.51%) BUSCO genes were identified as being full-length in the ‘Sanfensan’, *A. insularis*, and *A. longiglumis* assemblies, respectively. We also calculated the LTR assembly index (LAI) score for each of the genome assemblies using the LTR_retriever program (v2.8)[19] with a 3 Mb window size and a 1.5 Mb

sliding step. The results showed LAI scores of 18.34, 13.98, and 15.81 for the 'Sanfensan', *A. insularis*, and *A. longiglumis* genome assemblies, respectively, all of which meet the standard for reference quality (10< LAI <20)[20]. Finally, we aligned the raw ONT reads from 'Sanfensan', *A. insularis*, and *A. longiglumis* back to their corresponding genome assemblies using BWA with the default parameters; 99.87%, 98.62%, and 98.81% of the genomic regions of the 'Sanfensan', *A. insularis*, and *A. longiglumis* genomes were covered at more than 20× sequencing depth with ONT reads.

In addition, we compared the 'Sanfensan' assembly with the hexaploid oat consensus genetic linkage map constructed from 12 bi-parental populations representing 19 parents[21]. This map contains 21 linkage groups with 99,878 anchored markers. We first filtered these markers because many of them are redundant. For example, there are 30,995 tag-level (TL) haplotypes identified from GBS data from 4,661 oat lines, and 43,466 GBS-SNPs which are derived from these TL haplotypes when the TL haplotypes contain multiple SNPs; hence these TL haplotypes and GBS-SNPs share the consensus DNA sequences. In such cases, we retained only the TL haplotypes to avoid redundancy. In addition, 1,952 TL haplotypes were removed because these TL haplotypes and their derived GBS-SNPs mapped to different groups or mapped to positions separated by more than 10 cM. Finally, 45,738 marker sequences were searched against the 'Sanfensan' reference genome using BLASTn analysis. All hits with identity >85% and coverage rate >80% were retained.

1.9 Identification of centromeric regions

Previous studies reported that a family of sequences (CCS1) was located by *in situ* hybridization to the centromeres of many cereal species[22]. This repeat is also

concentrated in the centromeric regions in the A and D subgenomes of hexaploid oat [23,24]. In addition, Maugham *et al.*[10] reported a 159-bp long tandem repeat that aligns specifically with the putative centromere location in the C-genome diploid chromosomes. Hence, the chromosomes' centromeres of the three assembled oat genomes were identified and positioned based on the distributions of these two repeats. To this end, the locations of these two repeats in the three assembled genomes were determined by BLASTn analyses. All BLASTn bits with coverage <50% and identity <60% were filtered out. The proportions of these two repeats along each chromosome were then calculated using a non-overlapping sliding window of 1 Mb. The results showed (**Fig. 1a**) that CCS1 is concentrated in a narrow region on each of the A- and D-genome chromosomes, whereas no such concentration of the C-diploid centromeric-specific repeat was observed in the tetraploid and hexaploid C-genome chromosomes, suggesting that the centromeres of the C-genome chromosomes have largely diverged from those of the A and D chromosomes, and might have also undergone rapid changes after polyploidization events.

2. Genome annotation

2.1 Protein-coding gene annotation

Protein-coding genes were predicted using an evidence-based annotation workflow by integrating different sources of evidence.

Transcriptome-based evidence was generated with the following methods. First, full-length transcripts from PacBio sequencing were used to produce high-quality opening reading frame (ORF) predictions. For this purpose, raw PacBio transcriptome sequencing data was initially processed with the IsoSeq3 pipeline in SMRT Link (v8.0) (https://github.com/PacificBiosciences/IsoSeq). Briefly, the “ccs” command (--min-passes 1 --min-rq 0.8) was used to generate circular consensus sequences (CCSs), which resulted in 1,163,006 (2,476,793,041 bp), 726,902 (1,613,862,251 bp), and 374,567 (825,983,822 bp) CCSs for ‘Sanfensan’, *A. insularis,* and *A. longiglumis*, respectively. The "lima" and "refine" subprograms of IsoSeq3 were applied to identify the full-length, non-chimeric CCSs with the subsequent step of primer and poly-A tail sequence removal. These sequences were subsequently clustered using an iterative clustering and error correction (ICE) algorithm to obtain unpolished consensus isoforms, which were further polished by using the non-full-length reads and raw bam files with quiver parameters, resulting in a total of 1,150,752, 664,173, and 371,031 high-quality CCSs for ‘Sanfensan’, *A. insularis,* and *A. longiglumis*, respectively. The resulting high-quality CCSs were mapped to the reference genome using minimap2 (v2.18)[8] software with the default settings. Following this, “fusion_finder.py” and “collapse_isoforms_by_sam.py” scripts implemented in the cDNA_Cupcake (v24.3.0) software (https://github.com/Magdoll/cDNA_Cupcake) were subsequently used to filter out fusion genes and redundant sequences, which resulted in the retention of a

total of 53,812, 36,397, and 17,961 non-redundant isoforms for ‘Sanfensan’, *A. insularis*, and *A. longiglumis*, respectively. Finally, the non-redundant full-length transcripts were mapped to the reference genome assemblies using GMAP[25] with the default settings, and the resulting BAM files were used as the input for GeneMarkS-T[26] to determine the locations of potential intron-exon boundaries.

Second, a set of homologous proteins from other closely related species was employed as homology evidence using GeMoMa (v1.6.1)[27]. These species included *Avena atlantica*, *Avena eriantha*, *Brachypodium distachyon*, *Hordeum vulgare*, *Oryza sativa*, and *Triticum aestivum*.

De novo gene predictions were generated using AUGUSTUS (v2.4)[28]. For this purpose, an oat-specific AUGUSTUS gene model was trained using GeneMark-ET (v4.0)[29] with the following parameters: -max_intron max_intron -soft_mask soft_length -pbs -sequence=genome -ET=introns.gff. GeneMark-ET uses Iso-Seq evidence as training data and performs two rounds of iterative gene predictions to train model parameters. The 2,000 gene models with the highest scores were used as training data for AUGUSTUS. The resulting gene models were then used to predict the coding genes using AUGUSTUS (-gff3=on-hintsfile=hints.gff-extrinsicCfgFile= extrinsic.cfg-allow_hinted_splicesites=gcag,atac-min_intron_len=30-softmasking=1).

Finally, all gene predictions were integrated into a final gene annotation set using EVidenceModeler (v1.1.1)[30] (parameters: -segmentSize 1000000 -overlapSize 100000) after removing transposable element-related genes, pseudogenes, and noncoding genes using TransposonPSI (v1.0.0)[31] with the default settings. The results of the annotation process are summarized in **Supplementary Table 4**.

2.2 Functional annotation of gene models

Functional assignments for the predicted protein-coding genes were performed with BLAST by aligning the coding regions to sequences in public protein databases, including the NCBI nonredundant (NR) protein, Kyoto Encyclopedia of Genes and Genomes (KEGG), Eukaryotic Orthologous Groups of proteins (KOG), Gene Ontology (GO), and SwissProt databases. The putative domains and GO terms of the predicted genes were identified using InterProScan (v5.22)[32] with the default settings. A total of 103,773, 81,027, and 40,216 genes were functionally annotated for 'Sanfensan', *A. insularis* and *A. longiglumis*, respectively, comprising 88.41%, 90.04%, and 92.50% of the predicted gene models in each genome assembly (**Supplementary Table 5**).

2.3 Noncoding RNA prediction

Noncoding RNAs (ncRNAs), which include microRNAs (miRNAs), small nuclear RNAs, rRNAs, and regulatory elements, were identified using the Infernal (v1.1.2)[33] program to search against the Rfam database[34]. RNAmmer (v1.2)[35] (parameters: -S euk -m lsu,ssu,tsu –gff) was additionally used to predict rRNAs in more detailed subclasses. Transfer RNAs (tRNAs) were predicted using tRNAscan-SE (v2.0)[36] with eukaryotic parameters. miRNAs were predicted using miRanda (v3.0) (http://www.microrna.org). A total of 59,916, 40,282, and 15,706 ncRNAs were identified in the 'Sanfensan', *A. insularis* and *A. longiglumis* genomes, respectively.

2.4 Repetitive element annotation

Tandem repeats (TRs) in the genome assemblies were identified using GMATA (v2.2)[37] and Tandem Repeats Finder (v4.07b)[38] with the following parameters: 2 7 7

80 10 50 500 -f -d -h -r.

Species-specific *de novo* repeat libraries were constructed with the following steps: First, MITE-Hunter[39] software (parameters: -n 20 -P 0.2 -c 3) was used to identify miniature inverted TEs (MITEs). Then, LTR_FINDER (v1.05)[40] and LTR_harvest (v1.5.10)[41] were used for long terminal repeat (LTR) identification, and the results were processed with LTR_retriever (v2.8)[19] to generate an LTR library. Third, the TR soft-masked reference genome assemblies were hard-masked with both MITE and LTR libraries by using RepeatMasker (v1.331)[42] with the following parameters: nolow -no_is -gff -norna -engine abblast -lib lib, and other repetitive elements were identified *de novo* with RepeatModeler (v2.0.1)[43] (parameters: -engine wublast) (https://github.com/Dfam-consortium/RepeatModeler) and classified using TEclass[44] with the default parameters. Finally, the libraries obtained from MITE, LTR, and RepeatModeler were merged to generate the species-specific *de novo* repeat library, which was used along with the repetitive elements in Repbase (v19.06)[45] to annotate the genomes with RepeatMasker. The major TE-related repetitive elements annotated in each genome are summarized in **Supplementary Table 6**.

2.5 Pseudogene annotation

The pseudogenes in each species were identified using Pseudopipe[46]. Each of these pseudogenes was then aligned to the parent gene using MACSE (v2)[47] and only genes with a frameshift or nonsense mutation were considered to be candidate pseudogenes. The total number of pseudogenes in each assembled genome is given in **Table 1**, and their distributions on the chromosomes are visualized in **Extended Data Fig. 8c.**

3. Subgenome assignment, validation, and nomenclature

A reference-guided strategy based on genomic sequence similarity between the polyploid and diploid genomes was used to distinguish the subgenomes of *A. insularis* and ‘Sanfensan’. For the ‘Sanfensan’ subgenome assignments, we first split the sequenced *A. longiglumis* genome into 100 bp non-overlapping fragments that were subsequently aligned to the hexaploid ‘Sanfensan’ reference genome using BWA with the default settings. Uniquely mapped markers were retained. A syntenic block was generated when more than five fragments were consecutively distributed in a syntenic manner (distance between every two adjacent fragments of <200 kb). The results showed that the 21 chromosomes of ‘Sanfensan’ should be split into three groups each containing seven chromosomes; the group that had distinctly more uniquely mapped fragments was designated as the A subgenome and the group that had the fewest uniquely mapped fragments was designated as the C subgenome, because the C genome has largely diverged from the A genome as demonstrated by previous studies. The remaining group of seven chromosomes was accordingly assigned to the D subgenome (**Extended Data Fig. 4a, c)**. Similarly, the genome sequence of *A. insularis* was divided into 100 bp fragments and aligned to the hexaploid ‘Sanfensan’ reference genome. Most of the 100 bp fragments from the 14 chromosomes were uniquely mapped to chromosomes of ‘Sanfensan’ that were designated as the C and D subgenomes **(Extended Data Fig. 4b, d)**. These chromosomes were accordingly assigned to the tetraploid C and D subgenomes.

Two independent approaches were used to validate the subgenome assignments. First, trimmed Illumina short reads from *A. longiglumis* and *A. insularis* were individually mapped to the hexaploid ‘Sanfensan’ reference genome with BWA using

the default settings. The median depth coverage of the sliding windows (window size: 1 Mb, step size: 0.5 Mb) for *A. longiglumis* or *A. insularis* was calculated using the Mosdepth (v0.3.0)[48] program. The results showed that a much higher mapping depth was achieved for the hexaploid A subgenome chromosomes than the chromosomes of the other two subgenomes after mapping the *A. longiglumis* reads to the 'Sanfensan' genome, while the chromosomes assigned to the C and D subgenomes showed higher mapping depths than the A subgenome chromosomes after mapping the *A. insularis* reads to the 'Sanfensan' reference genome (**Extended Data Fig. 3e, f**). Second, the abundances and distributions of two types of satellite repeats, As120a and Am1, in all three assembled genomes were determined by BLASTn analyses. As120a and Am1 are DNA repeats that have been shown to be specifically enriched in the hexaploid A[49] and C[50] chromosomes, respectively. We observed that the A genome-specific repeat As120a is over-represented in the seven *A. longiglumis* chromosomes and the seven chromosomes of the hexaploid 'Sanfensan' that are assigned to the A subgenome. Similarly, the C genome-specific repeat Am1 is more abundant in the seven *A. insularis* chromosomes and the seven 'Sanfensan' chromosomes that are assigned to the C subgenome (**Fig. 1**). All these analyses support the subgenome assignments for tetraploid *A. insularis* and hexaploid 'Sanfensan'.

The nomenclature system for wheat chromosomes was adopted for naming the homologous groups (1-7) of 'Sanfensan'. For this purpose, whole-genome protein sequences and gene positions from hexaploid bread wheat (IWGSC RefSeq v1.1) were retrieved from the GrainGenes database (https://urgi.versailles.inrae.fr/download/iwgsc/IWGSC_RefSeq_Assemblies/v1.1/). If a gene had more than one transcript, only the longest transcript was retained as the representative sequence. The synteny

between the bread wheat and 'Sanfensan' genomes was analyzed using the MCScanX (git-97e74f40)[51] program with the default settings. We found that all chromosomes except the putative 4C and 7C could be named unequivocally based on the synteny results. Both the putative 4C and 7C chromosomes are heavily rearranged, but the share a large fragment with the wheat group four chromosomes. The same strategy was used for naming the chromosomes of the OT3098 v2 reference genome (https://wheat.pw.usda.gov/jb?data=/ggds/oat-ot3098v2-pepsico), which also showed that these two C chromosomes are heavily rearranged. To solve this problem, the International Oat Nomenclature Committee (IONC) was reconvened, and the committee found that when the syntenic plots are reduced to only the core centromere regions, one of the two C chromosomes was inherited from the chromosome 4 ancestor; therefore, it was named 4C. Likewise the centromere from the other one was inherited from the chromosome 7 ancestor. This nomenclature system was approved by the IONC and used for the other hexaploid oat OT3098 v2 reference genome. To make the nomenclature system consistent among different oat genomes, the nomenclature system used in OT3098 v2 was also adapted to naming and orienting the chromosomes of our genome assemblies.

4. Phylogenomics and comparative genomic analyses of cereal crops

4.1 Phylogenetic tree construction and divergence time estimation

To understand the evolutionary position of *Avena* species within the grass family, predicted protein sequences of five *Avena* species and 11 other grass species mainly representing the Pooideae subfamily of the grass family (**Supplementary Table 7**) were used to identify the conserved single copy genes which were then used to infer the phylogenetic relationships of these grass species. To this end, proteins of these species with sequence length <50 amino acids were first removed, and for genes with alternative splice variants only the longest transcript was selected. Then the single copy orthologous gene clusters from these species were identified by OrthoFinder (v2.2.7)[52]. In this step, the subgenomes of the polyploid species were handled as independent taxa. In total, 2,237 single copy orthologous gene clusters shared by all 23 subgenomes from 16 species were identified. For each of these single copy gene clusters, protein sequences of the homologous genes were aligned using MUSCLE (v3.8.31)[53] with the default parameters, and the alignments were CDS-back-translated into CDSs using an in-house perl script (Code was archived on Zenodo with the DOI https://doi.org/10.5281/zenodo.6622160[54]). The conserved CDS alignments were extracted by Gblocks (v0.9b)[55], and the retained CDS alignments of each family were used for further phylogenetic analyses.

For phylogenetic tree construction, the CDS alignments of each single-copy gene family were concatenated to generate a supermatrix of 2,397,150 unambiguously aligned nucleotide positions, and 764,013 4DTv (4-fold degenerate transversion) sites were then extracted from these supergenes and subjected to RAxML (v8.2.7)[56] analysis to generate a maximum likelihood tree with the GTR+I+Γ model.

Divergence times were estimated under a relaxed clock model using the MCMCTree package of PAML (v4.7)[57] with the "Independent rates model (clock=2)" and "JC69" (Jukes-Cantor 1969) model in the PAML (v4.7) package. Considering that evolutionary rates vary at the different codon positions, the three codon positions of a concatenated supergene were treated as three different partitions. The MCMC process was run for 6,000,000 iterations after a burn-in of 2,000,000 iterations. We ran the program twice for each data type to confirm that the results were similar between runs. The chronogram was produced using FigTree (v1.4.0) (http://tree.bio.ed.ac.uk/) with the first run (**Fig. 2a**).

4.2 Gene family analysis

Gene families were defined using an automated phylogenomics approach incorporating the predicted protein sequences from the 23 subgenomes from 16 species with the subgenomes of the polyploid species separated. Pairwise sequence similarities between all input protein sequences were calculated using BLASTp with an E-value cut-off of 1E-05 followed by the removal of low-quality hits (identity <30% and coverage <30%). The OrthoFinder (v2.2.7) program was used to cluster the alignment results into family groups with the default settings. A total of 834,539 coding sequences from these 23 (sub)genomes were clustered into 42,708 gene families. 6,042 clusters contained sequences from all 23(sub)genomes (Fig. 2b). Expanded and contracted gene families for each (sub)genome were identified by comparing the cluster size differences between the ancestor and each (sub)genome using CAFÉ (v5.2.1)[58]. A random birth-and-death model was employed to evaluate the changes in gene families along each lineage of the phylogenetic tree. A probabilistic graphical model (PGM) was used to calculate the probability of

transitions in each gene family from parent to child nodes in the phylogeny. Using conditional likelihoods as the test statistics, we calculated the corresponding *P*-values of each lineage and a *P*-value <0.05 was used as the cutoff to determining the significance of family size change (**Supplementary Table 8**). The results showed that the numbers of gene families in each *Avena* genome or subgenome are comparable to those in other diploid grass species.

Genes that were exclusively found in all species belong to the tribes Aveneae, Lolieae and Triticeae were defined as specific genes for these groups, which resulted in 1,608, 3,670, and 1,070 specific gene families respectively (**Fig. 2b**). GO terms for the Aveneae-specific genes were identified using the R package from Bioconductor (http://bioconductor.org/packages/release/bioc/html/clusterProfiler.html). Significant terms were reported up to an adjusted *P*-value ≤0.05 (**Supplementary Table 10**).

4.3 Karyotype evolution

Rice was identified as the most slowly evolving species and has 12 chromosomes, most of which closely resemble the post-ρ AGK[59]. We used the rice genome as an ancestral reference to investigate karyotype origins and used the barley genome as closely reference as suggested to compare chromosome evolution with wheat. The gene homeologs of bread wheat and four *Avena* species (*A. eriantha, A. longiglumis, A. insularis*, and 'Sanfensan') were aligned with the rice or barely genome. Syntenic blocks that were defined based on the presence of at least three syntenic gene pairs were identified using the MCScanX package. These syntenic blocks were then used to deduce the homologous relationships between rice or barely genes and the homologous gene sequences from *Avena* species and wheat (**Supplementary Table 11**).

5. The evolution and allopolyploidization history of oat

5.1 Whole-genome sequencing-based analyses

5.1.1 Plant material

To clarify the evolutionary history of hexaploid oat, 14 *Avena* accessions, representing all extant diploid and tetraploid genomes, were chosen for whole-genome sequencing. These included As-, Al-, Ad-, Ac-, Cv-, and Cp-genome diploids, AB- and CD-genome tetraploids and ACD-genome hexaploid species. Detailed information on these species, including their genome constitutions, accession numbers, and geographical origins, is given in **Supplementary Table 1**.

5.1.2 Whole-genome sequencing

For the sequencing of the selected accessions, DNA was isolated from young leaf tissue of a single plant using the Qiagen DNeasy Plant Mini Kit and 400-bp paired-end (PE) libraries were prepared. Sequencing was conducted on an Illumina HiSeq X-Ten sequencer at the Genome Centre of Grandomics (Wuhan, China) (**Supplementary Table 1)**. Raw data were subjected to quality control. Low-quality reads were removed if they met any of the following criteria: (1) >50% of the bases having a quality score <Q30; (2) the proportion of unidentified (N) nucleotides was >5%; (3) reads overlapping >10 bp with the adapter sequence, allowing <2 bp mismatch. Summary statistics for whole-genome sequencing of the *Avena* accessions are shown in **Supplementary Table 1**.

5.1.3 Identity plots

For each accession that was subjected to whole-genome sequencing, approximately 1× coverage of clean short paired-end reads were randomly extracted from the resequencing data. These reads were then mapped to the repeat hard-masked

‘Sanfensan’ reference genome using BWA with the default parameters. Uniquely mapped reads were extracted using SAMtools (v1.9) (samtools view -bS -f 3 -q 10). The best hit for each read was retained when the BLASTn score was 15 greater than that of the suboptimal hit and the query coverage was >60 bp. The average identity over a sliding window of 20 Mb was calculated and plotted against the chromosomes of the ‘Sanfensan’ assembly with a step size of 1 Mb.

5.1.4 Variant calling

Given the different ploidy levels across these accessions, a specific pipeline was adapted for variant calling. For the diploid species, the cleaned reads were mapped individually to the A, C, and D subgenomes of the ‘Sanfensan’ reference genome using the BWA program with the default settings. For the CD-genome tetraploids, cleaned reads were first aligned to the tetraploid *A. insularis* reference genome with BWA. Reads that were uniquely mapped to the subgenomes of the tetraploid were retrieved based on the BAM files. The subgenome-specific reads were then mapped against each subgenome of the ‘Sanfensan’ genome. For the hexaploid accessions, cleaned reads were first mapped to the ‘Sanfensan’ reference genome, reads that were uniquely mapped to the subgenomes of the hexaploid were retrieved based on the BAM files, and these subgenome-specific reads were then respectively aligned to the subgenomes of the hexaploid ‘Sanfensan’ reference genome. Because of the lack of a reference genome for the B subgenome, the sequencing data of the tetraploid AB species could not be divided based on their subgenome source, so they were directly mapped onto each of the hexaploid ‘Sanfensan’ subgenomes. Duplicated reads were removed using SAMtools. The genomic variants were identified using the HaplotypeCaller module in GATK (v4.1.9.0), and SNPs that met any of the following

criteria were further discarded: (1) SNPs with quality score <50, QD score <2, FS score >60 or MQ score <40; (2) SNPs with more than two alleles; (3) SNPs at or within 5 bp from any InDels; (4) Genotypes with extremely high (greater than three-fold average depth) or extremely low (less than one-third average depth) coverage were assigned as missing sites; (5) SNPs with a minor allele frequencies (MAF) < 0.05; and (6) SNPs with missing sample rate >0.3.

5.1.5 Phylogenetic tree construction using SNPs

SNPs in syntenic regions across the A-, C-, and D-subgenomes for each accession were isolated and used for maximum-likelihood (ML) tree construction, which was performed using the RAxML software with the following parameters: --all –model GTR+ASC_LEWIS –tree pars{10} –bs-trees. Bootstrap support was set at 200 replicates. To further explore the phylogenetic relationships of the A-, C-, and D-lineages, ML trees based on the A-, C-, and D-type SNPs were constructed. In this case, only accessions or subgenomes closely related to the A, C, and D subgenomes were included in A-, C-, and D-type ML tree constructions.

5.2 Transcriptome sequencing-based analyses

5.2.1 Plant growth and RNA isolation and sequencing

All diploid accessions that were subjected to whole-genome sequencing were included in the transcriptome analysis. Plants were grown in the greenhouse or the field to different growth stages. Seven sample types from each line, (as described in section 1.5, PacBio Iso-Seq), were collected for RNA extraction. RNA was extracted using a Qiagen RNA isolation kit and RNA quality was accessed by 0.75% agarose gel electrophoresis and on an Agilent 2100 Bioanalyzer. High-quality RNA preparations from the seven sample types from each accession were mixed in equal

amounts. Sequencing libraries were prepared using the MGIEasy RNA Directional Library Prep Kit (BGI, China) according to the manufacturer's protocol, and 400-base paired-end (PE) sequencing was performed using an MGISEQ-2000 instrument at the Genome Centre of GrandOmics (Wuhan, China) (**Supplementary Table 1**).

5.2.2 Transcript assembly and CDS prediction

Raw MGI sequencing reads were filtered using the following steps: read pairs with adapter contamination, read pairs with N contents >3%, and read pairs with >20% low-quality bases (Phred Q scores <20) were first removed. Reads with potential low-quality regions were then trimmed with Trimmomatic (v0.40)[7]. Reads with a Q score <15 at both ends were also trimmed off, and reads containing 3' or 5' ends with an average quality score dropping below Q20 in a 4-bp sliding window were trimmed. Finally, all reads of <32 bp were excluded to obtain clean data for further analyses. The clean reads were assembled *de novo* using Trinity (v2.0.3)[60] with the default parameters. The CDSs were predicted using TransDecoder (v5.5.0) (https://github.com/TransDecoder/TransDecoder).

5.2.3 Phylogenetic tree construction and divergence time estimation

The proteomes from these diploid accessions were subjected to gene family analysis against *Hordeum vulgare* (barley) protein sequences using OrthoFinder2, and single-copy gene families that were conserved in all of the species were retained for further study. The protein sequences from each conserved gene family were aligned using MUSCLE (v3.8.31) with the default parameters, and the alignments were CDS-back-translated into CDSs from the corresponding protein alignments. The same methods described in section 4.1 were used for phylogenetic tree construction and estimation of divergence times.

5.3 Organelle-based analyses

The chloroplast genomes of *A. longiglumis*, *A. insularis*, 'Sanfensan', and the other taxa subjected to whole-genome sequencing were assembled using high-quality short paired-ended reads (**Supplementary Table 13**) with NOVOPlasty (v3.7) (https://github.com/ndierckx/NOVOPlasty); the chloroplast DNA sequence from *A. murphyi* (GenBank Accession: NC_044174.1) was used as the reference. We downloaded 25 additional *Avena* chloroplast genomes (**Supplementary Table 14**) to obtain a more comprehensive dataset. Multiple sequence alignments were performed using MUSCLE, and the informative sites were used for the construction of a phylogenetic tree, with the cpDNA of *Triticum aestivum* as the outgroup. All of these analyses were performed with RAxML (v8.2.7) with the following parameter settings: -m GTRGAMMAI -N 100 -f a -k -d -p 12345 -x 12345).

Divergence times were estimated under a relaxed clock model using the MCMCTree program in the PAML (v4.7) package. The "Independent rates model (clock=2)" and "JC69" models in the MCMCTree program were used. The MCMC process was run for 6,000,000 iterations after a burn-in of 2,000,000 iterations. We ran the program twice for each data type to confirm that the results were similar between runs. The chronogram was visualized using FigTree (v1.4.0) with the first run.

6. Subgenome evolution

6.1 Chromosome rearrangement

6.1.1 Synteny analysis

Synteny among the subgenomes of *A. insularis* and ‘Sanfensan’ was individually analyzed by plotting the positions of homoeologous pairs in the subgenome pairs within the context of the 14 (*A. insularis*) and 21 (‘Sanfensan’) chromosomes using Circos (v0.69-9)[61] (**Extended Data Fig. 5**). For this purpose, homoeologous gene pairs within the tetraploid and hexaploid genomes were determined based on reciprocal BLAST. Genes exhibiting best bi-directional hits between homoeologous segments (*e.g.*, 1A to 1D and 1C to 1D) and between homoeologous subgenomes were considered as homoeologs. Syntenic blocks were then identified used MCScanX (>=3 genes per block). Similar approach was used to identify orthologous genes between the different subgenomes of hexaploid ‘Sanfensan’ and its ancestral diploid and tetraploid genomes. The syntenic blocks shared between the ‘Sanfensan’ subgenomes and its ancestors (tetraploid *A. insularis* and the diploid *A. longiglumis*) were identified using MCScanX (>=3 genes per block) and were visualized using Circos (v0.69-9) (**Fig. 1**).

To explore broad-scale structural variations after polyploidization, we used ‘Sanfensan’ to perform *in silico* painting of the *A. insularis* and *A. longiglumis* genomes using the method described previously[62]. In brief, the ‘Sanfensan’ genome was divided into 100 bp non-overlapping fragments that were then aligned to the concatenated repeat hard-masked genomes of *A. insularis* and *A. longiglumis* using BWA with the default settings. The uniquely-mapped fragments with alignment lengths >50 bp in the target genome were retained. We then processed the fragments

on each chromosome by requiring at least five consecutive fragments supporting homology to the same 'Sanfensan' chromosome. We consolidated each group of five consecutive potential fragments into one confirmed block. These confirmed blocks with length <2 Mb were further consolidated into superblocks (**Fig 4a, bottom**). A similar painting analysis was performed by painting 100 bp fragments from *A. insularis* onto concatenated genomes of *A. longiglumis* and *A. eriantha* (**Fig. 4a, top**). The total DNA sequence length from each chromosome of 'Sanfensan' and *A. insularis* that was uniquely mapped onto each chromosome of their putative ancestors was counted (**Supplementary Tables 15-16**). To further explore the genomic exchanges between *A. insularis* and 'Sanfensan' that occurred after polyploidization, clean, short paired-end reads from the Cp-genome diploid *A. eriantha* and the Al-genome diploid *A. longiglumis* were individually mapped onto the *A. insularis* and 'Sanfensan' reference genomes using BWA. The single-base depth coverage of properly paired reads from the *A. longiglumis* and *A. eriantha* mapping results was calculated using the Mosdepth program and plotted along each chromosome of the reference genome (**Fig. 4b, Extended Data Fig. 6a-c**). A similar analysis was performed by aligning reads from *A. insularis* to the 'Sanfensan' reference genome **(Extended Data Fig. 6d**).

6.1.2 Fluorescence in situ hybridization (FISH)

FISH was used to validate the observed C-to-D and C-to-A intergenomic translocations in the *A. insularis* and 'Sanfensan' genomes, as well as the large inversion between the tetraploid and hexaploid 3C chromosomes. For the validation of the C/D and C/A translocations, FISH analysis was performed using the A- and C-genome-specific repeat As120a, Am1 as the probes, whereas to validate the large

inversion between the tetraploid and hexaploid 3C chromosomes, we used two additional oligonucleotide probes, Oligo-5SrDNA and Oligo-6C343[24]. A previous study[24] showed that both the Oligo-5SrDNA and Oligo-6C343 probes could give clear hybridization signals on the long arm of the hexaploid chromosome 3C. Searching these two sequences against the 'Sanfensan' reference genome showed that Oligo-5SrDNA and Oligo-6C343 are also enriched in the 3C chromosome of 'Sanfensan', concentrating in the two regions between 471-472 Mb and 519-520 Mb, respectively, which are located in the flanking regions of one of the breakpoints of the inversion. Therefore, the signal positions of these two probes in the tetraploid and hexaploid chromosome 3C could be used to validate the inversion.

The FISH probe was prepared as described previously[63]. All nucleotide sequences of the FISH probes were listed in **Supplementary Table 17.** The metaphase chromosome preparation method was that used in a previous experiment[63] with some modifications. In brief, seeds of *A. insularis* and 'Sanfensan' were imbibed in distilled water for 18 h at 25°C in the dark and then placed in Petri dishes with two layers of moist filter paper. The germinated seeds were transferred to a cabinet at a temperature of 4°C to synchronize cell division and allow the accumulation of metaphase plates. Root tips were harvested when they reached a length of 1.5-2.0 cm and were pre-treated in 1.0 MPa nitrous oxide gas for 3 h followed by fixation using glacial acetic acid for 20 min. The apical meristem was extruded from the fixed root tip and digested with 2% cellulase and 1% pectinase for 2 h. The digested apical meristem was squashed in a drop of 60% acetic acid, and the resulting suspension was dropped onto a clean glass side.

FISH analysis was performed as described by Fu *et al.*[64]. Briefly, air-dried slides

were fixed for 10 min with 4% (w/v) paraformaldehyde and then immersed in 2× saline sodium citrate (SSC) for 10 min. After dehydration in an ice-cold ethanol series (75%, 95%, and 100%) for 5 min in each concentration, the slides were air dried and then subjected to denaturing at 80°C for 2 min in deionized formamide (60 μl per slide), followed by dehydration in 75%, 95%, and 100% ethanol at -20°C for 5 min each before air drying again. A 10 μl aliquot of a hybridization mixture containing 0.5 μl of the FISH probe, 4.75 μl of 2× SSC, and 4.75 μl of 1×X TE was applied to each slide, and the slides were then incubated for 2 h at 37°C. The slides were next counterstained with DAPI (4′,6-diamidino-2-phenylindole) and mounted with Vectashield mounting medium (Vector Laboratories, Inc., Burlingame, CA, USA). Digital images were captured using an Olympus BX-51 epifluorescence microscope equipped with a Photometric SenSys Olympus DP80 CCD camera (Olympus, Tokyo) and processed using Photoshop (v7.0) (Adobe Systems Incorporated, San Jose, CA) (**Fig. 4c-d, Extended Data Fig. 6e-f**).

6.1.3 Functional enrichment of genes involved in translocations

Chromosomal rearrangements are important mechanisms that drive evolution and environmental adaptation, as well as contributing to phenotypic diversity[65]. In oat, the well-documented 1C/1A (previously referred as 1C/17A) translocation has been demonstrated to be associated with the division of cultivated oat into the two species *A. sativa* L. and *A. byzantina* K. Koch and the differences in crown freezing tolerance and winter field survival[66]. To further understand whether genes within these translocation regions are correlated with genome evolution or environmental adaptation, breakpoints of the seven large (>40 Mb) translocations including two D/C (D-to-1C, D-to-4C) and four C/D (C-to-3D, C-to-4D, C-to-5D, and C-to-6D)

translocations observed in the tetraploid, and the 1C/1A translocation observed in the hexaploid were identified by manually checking the values of the coverage depths, and genes within the translocation regions were extracted for functional enrichment analysis. Significant terms were reported up to an adjusted *P*-value of ≤0.05 (**Supplementary Tables 18-19**).

6.2 Subgenome contents

6.2.1 k-mer distribution

The 31-mer frequencies in the sliding windows (window size: 1 Mb, step size: 0.5 Mb) of the Al, CD, and ACD genome assemblies were counted using Jellyfish[2], and the highest frequencies in each window were plotted along the chromosomes (**Fig. 1**).

6.2.2 Genome size variation analyses

The C subgenome (4.09 Gb) is ~20% larger in size than the A (3.35 Gb) and D (3.22 Gb) subgenomes in the 'Sanfensan' genome (**Table 1**). Similar magnitudes of genome size variation were observed in the subgenomes of the tetraploid species *A. insularis* (**Table 1**). These results are consistent with previous flow cytometry analyses which reported that the genomes of the C-genome diploids are ~15% larger than the genomes of the A-genome diploids[67]. Comparison of the assembled genome sizes of the three subgenomes of the hexaploid with the tetraploid and diploid A genomes revealed that the C and D subgenomes experienced relatively small genome size variations (tetraploid C vs. hexaploid C, 4.02 Gb vs. 4.09 Gb; tetraploid D vs. hexaploid D, 3.13 Gb vs. 3.22 Gb) after hexaploidization. In contrast, the hexaploid A subgenome showed a 9.70% contraction (diploid A vs. hexaploid A, 3.71 Gb vs. 3.35 Gb) compared to its A genome diploid ancestor. Likewise, the D subgenome of the tetraploid is 15.63% smaller after tetraploidization if the D-genome diploid ancestor

had a similar genome size to the A-genome diploids. Compared to the diploid C genome with an assembled genome size of 3.77 Gb[10], the tetraploid C subgenome showed a 6.63% size expansion; however, caution is advised when considering this conclusion since the size of the diploid C genome assembly was far smaller than the genome size estimated by k-mer frequency (4.17 Gb)[10] and flow cytometry[67].

6.2.3 Repetitive DNA comparison

The overall repetitive DNA proportions of the three assembled genomes were similar, ranging from 86.83% to 87.11%. The most commonly identified repetitive elements were long terminal repeat retrotransposons (LTR-RTs), accounting for 70.63% to 74.83% of the genomes, with most of them classified in the *Gypsy* and *Copia* superfamilies (**Supplementary Table 6**). The third and fourth most abundant repetitive elements were DNA transposons and non-LTR retrotransposons (LINEs), respectively. Overall, the C subgenome has the highest content of TE sequences with 3.61 Gb in total length compared with the A and D subgenomes with 2.88 Gb and 2.76 Gb, respectively, accounting for almost all of the size differences between them. Likewise, approximately 0.82 Gb (92.13%) and 0.34 Gb (94.44%) of the genome size differences between the tetraploid C and D subgenomes, and the diploid A genome and hexaploid A subgenome, were contributed by TE abundance (**Supplementary Table 6**).

6.2.4 Gene content comparison

A total of 120,769 gene loci were predicted in the 'Sanfensan' genome assembly; of them, 36,283 were assigned to the C subgenome, a number that is 12.43% and 12.85% less than the gene loci in the A (41,433) and D (41,633) subgenomes, respectively. The remaining 1,420 gene loci were not assigned to any of the subgenomes.

Compared to the diploid A and tetraploid D and C (sub)genomes with 43,477, 43,911, and 43,243 genes, all three hexaploid A, D, and C subgenomes showed contractions of 4.70%, 5.19%, and 16.10%, respectively, in their gene contents. In contrast, the numbers of gene loci in the two tetraploid subgenomes were similar (C vs. D, 43,243 vs. 43,911). Compared to the diploid A and C genomes with 43,477 and 47,361 genes[10], the tetraploid D and C subgenomes showed a 1.00% expansion and 8.69% contraction in their gene contents, respectively. However, the number of genes lost from the tetraploid C subgenome might be an overestimate owing to the different pipelines used for gene annotation or a relatively limited completeness of the C-genome diploid assembly (N50 = 1.4 Mb).

6.2.5 Gene loss and retention

To measure the effects of polyploidization on gene loss and retention, we compared the changes in gene family sizes in the hexaploid subgenomes and the diploid genomes of *A. longiglumis* and *A. eriantha* based on the gene families identified by the OrthoFinder program. In this case, only clusters containing at least one gene from *A. longiglumis* and *A. eriantha* and at least one hexaploid oat gene were considered. The numbers of genes identified in families of different sizes were compared between each diploid genome and each of the subgenomes of hexaploid oat. The regression fit was calculated using the lm function implemented in R (parameter span=1) using only gene clusters with up to 10 members in the diploid species. The results showed that all three subgenomes of the hexaploid have undergone gene losses, with more gene losses occurring in the C subgenome (**Extended Data Fig. 8e-f**).

To further estimate the loss and retention rates of these conserved (single copy) genes after polyploidization, we performed presence and absence variation (PAV)

analyses with the A- and C-genome diploids as the references. For this purpose, single copy genes present in both the A-genome (*A. atlantica* and *A. longiglumis*) and C-genome (*A. eriantha*) diploid species were identified based on the OrthoFinder results, and the PAVs for each of the subgenomes of the tetraploid and hexaploid were counted and plotted with the chromosomes of the A-genome diploid *A. longiglumis* as the reference. A total of 14,624 one-to-one orthologues were identified for *A. longigulus*, *A. atlantica*, and *A. eriantha*. Of these genes, 1,618 and 1,315 have been lost from the C and D subgenomes of the tetraploid *A. insularis*, and 790, 2,367, and 1,079 have been lost from the A, C, and D subgenomes of the hexaploid 'Sanfensan'.

6.2.6 *Ka/Ks* analysis

Among the genome assemblies for *Hordeum vulgare*, the A- and C-genome diploid progenitors *A. longiglumis* and *A. eriantha*, and the subgenomes of *A. insularis* and 'Sanfensan', we fetched 7,353 one-to-one orthologous gene sets from the OrthoFinder results, and these were used for the nonsynonymous (*Ka*) and synonymous (*Ks*) rate calculations. For this analysis, the orthologous gene pair list was used as the input, and the protein sequences from each gene pair were aligned using MUSCLE. PAL2NAL (v14)[68] was used to convert the peptide alignment to a nucleotide alignment, and the *Ka* and *Ks* values were computed between the gene pairs using Codeml from PAML (v4.7) in free-ratios mode. All estimates with *Ks* <0.01 were excluded from the analysis. The average *Ka*/*Ks* values of all genes were used to represent the *Ka*/*Ks* values for the corresponding subgenomes. The significance of the differences in *Ka/K*s values between genomes (subgenomes) was estimated using the Wilcoxon rank-sum test for non-normal distributions in R (**Extended Data Fig. 9**).

6.3 Subgenome dominance

6.3.1 Plant materials and transcriptome sequencing

RNA was isolated from seven sample types from ‘Sanfensan’, including seedlings, flag leaves, and panicles at different developmental stages (described in section 1.5). Each type of RNA sample was sequenced with three biological repeats on an MGISEQ-2000 instrument. To further understand the transcriptional responses of genes in the different subgenomes of ‘Sanfensan’ under abiotic stress, seedlings of ‘Sanfensan’ were exposed separately to heat, cold, drought, waterlogging, alkalinity, and salt. For the abiotic treatments, oat plants were first grown in well-watered conditions in a growth chamber for 14 d at 20°C with a 12 h light/dark photoperiod, and the plants were then either left in these growth conditions as controls or transferred to other growth chambers for the stress treatments. For cold treatment, the plants were grown in a growth chamber at 4°C, while for heat treatment, the plants were grown in a growth chamber under a 12 h light/10 h dark cycle at 37°C (light) and 32°C (dark). For the drought and waterlogging treatments, the plants were carefully transferred to other pots containing either 10% PEG6000 or muddy soil. For the alkaline and salt treatments, water was replaced by 6 mmol/L alkaline solutions (Na_2CO_3:$NaHCO_3$ = 1:1) or a 40 mmol/L salt solution ($NaCl$:Na_2SO_4 = 1:1), respectively. One week after all the treatments were completed, the seedlings were harvested with three repeats for each treatment and used for RNA isolation. The same methods described in section 5.2 were used for construction and sequencing of the RNA sequencing libraries.

6.3.2 Quantification of gene expression levels

The paired-end MGI reads from the RNA samples described above were subjected to

quality trimming using Trimmomatic (v0.40) with the default settings and aligned to the gene models with HISAT2 (v2.2.1)[69] software with the default parameters. Gene expression levels were quantified using the HTseq (v0.9.1)[70] program with the 'Sanfensan' gene models as the references. Expression levels were quantified as transcripts per million (TPM) values.

6.3.3 Analysis of homoeologous gene expression

Differences in the expression patterns of 12,225 homoeologous gene sets (triads) in 'Sanfensan' were analysed to test for the presence of subgenome dominance, a striking whole-genome feature that is common in polyploids. For this purpose, the raw expression values (TPM values) of these triplets from seedlings, flag leaves, panicles at different developmental stages, and seedlings under six abiotic stresses were transformed by adding 1 and taking the common logarithm, and the expression matrix was subjected to two-dimensional hierarchical clustering using the correlation distance and the average linkage method to form clusters (**Fig. 4f**). The differentially expressed orthologous genes (DEOGs) between the different subgenome pairs were defined as gene triplets with a pairwise log2-fold change >0.5 and an adjusted *P*-value <0.05 (**Supplementary Table 21**). The expression patterns of these DEGOSs were visualized in the heatmap using the heatmap.2 command in the R package gplots (**Fig. 4g)**.

6.3.4 Relationship between gene expression and TE-density

To test whether the density of nearby TEs is correlated with gene expression levels, as reported in previous studies[71,72], we calculated the TE densities in the sequence regions 5 kb upstream and 5 kb downstream, both separately and together, for each gene from the 12,225 triads. The results revealed that the homoeologs from the C

subgenome of ‘Sanfensan’ had a higher TE density than those from the A and D subgenomes (**Extended Data Fig. 10**). The 12,225 genes in each subgenome were equally divided into four groups according to their TE densities near genes. The significances of the differences in the mean values of the gene expressions between each group were estimated using the Student’s *t*-test.

6.3.5 Characterization of alternative splicing variants

Data from both Iso-seq and RNA-seq was used to identify alternative splicing (AS) events in ‘Sanfensan’. To this end, transcripts from the RNA-seq data were first assembled using the StringTie (v2.2.0)[73] program with the default parameters based on the bam files generated from HISAT2, and these were then integrated with the Iso-seq transcript assemblies with SQANTI3 (v5.0)[74]. The number of AS events in each gene locus was counted using SQANTI3 with the default settings.

In total, alternative splicing variants were identified in 40,687 genes (3.38 transcripts per spliced gene), accounting for ~33.69% of the annotated genes in the ‘Sanfensan’ genome. Comparing the AS events in each subgenome, we observed that there were more spliced genes in the A (14,065) and D (14,049) subgenomes than in the C (12,167) subgenome. Furthermore, we examined the AS events of the 12,225 homeologous triads, as well as in the balanced and dominant genes in each of the subgenomes. The significance of the mean number of AS transcripts was determined using Student’s *t*-test (**Extended Data Fig. 10a).**

7. Genes related to agronomic traits

7.1 Identification of R-genes

To identify the putative R-genes present in five *Avena* species including our three assembled genomes and two previously reported diploid oat genomes (*A. atlantica* and *A. eriantha*), the domains of each gene model from these genomes were first annotated using Interproscan (v5.22)[32] by searching the Pfam and Gene3D databases. The proteins with the NB-ARC-domain (Pfam PF00931) and RPW8 domain (PF05659) were extracted as candidate R-genes. These candidates were also annotated with the CC (coiled-coil) domain using DeepCoil (v2.0.1). Finally, to obtain non-canonical R genes, we aligned the entire proteome of these *Avena* species to the manually curated R genes from the PRGdb4.0[75] database using BLASTp with an E-value cutoff of 1E-5, and the top hits with at least 30% query and subject coverage were retained. R-genes were grouped into clusters when they were not interrupted by more than eight other ORFs encoding non-R proteins.

A total of 1,269 R-genes were identified in the 'Sanfensan' genome, versus 971 and 406 R-genes in the genomes of *A. insularis* and *A. longiglumis*, respectively. These genes mainly clustered in high numbers in the distal regions of chromosomes. The chromosomes with most R-genes in the A, C, and D subgenomes were 1A (105), 6C (78), and 4D (109), respectively. Similar distribution patterns were observed for R-genes in the *A. insularis* and *A. longiglumis* genomes. Compared to the tetraploid C and D subgenomes and the diploid A genome with 437, 508, and 406 R-genes, respectively, the hexaploid C (302) and D (441) subgenomes had 30.89% and 13.19% contraction in the number of R-genes, while the R-genes in the hexaploid A (438) subgenome showed a 7.88% expansion.

Crown rust, caused by the fungus *Puccinia coronata* Corda f. sp. *avenae* Eriks, is the most widespread and damaging disease of oats, and the pathogen can cause severe damage in all regions where oat is cultivated[76]. At present, approximately 100 *P. coronata* resistance (*Pc*) genes have been reported[77], and some of them have been mapped using molecular markers (**Supplementary Table 22**). To understand whether these R-genes are correlated with the mapping positions of the known crown rust QTLs, DNA marker loci (**Supplementary Table 22**) that co-segregate with or flank the known crown rust resistance genes were mapped to the 'Sanfensan' genome assemblies by BLASTn analyses. Both the physical distributions of the R-genes and the crown rust QTLs on the chromosomes of hexaploid oat are displayed in **Fig. 5b**.

7.2 Identification of candidate genes for the hulless trait

7.2.1 Plant materials

A total of 659 diverse oat lines were selected for genotyping-by-sequencing (GBS) analysis. These lines came from three major sources: (1) Chinese oats including 128 hulless and 15 hulled cultivars, (2) a set of 145 oat lines nominated by breeders from North America and Europe, and (3) a set of 371 diverse lines collected from 52 countries or districts (**Supplementary Table 23**).

7.2.2 DNA isolation, library construction, and DNA sequencing

Genomic DNA of each accession was isolated from leaf samples of five one-week old seedlings using the Plant Genomic DNA kit (Catalog number DP201101X; Tiangen, China). The purity and concentration was checked using a spectrophotometer (Implen NanoPhotometer; www.implen.de) and a Qubit Fluorometer (Life Technologies, CA, USA). The reference 'Sanfensan' genome was used to predict the patterns of restriction enzyme digestions across the reference genome and to select the best

enzyme combination for DNA digestion. Based on this pilot experiment, the restriction enzymes *MseI* and *EcoRI* were chosen for genomic DNA digestion.

For library construction, genomic DNA was digested at 37°C with *MseI* and *EcoRI* (New England Biolabs, Ipswich, MA USA), and ligated by adding T4 DNA ligase (NEB), ATP (NEB), and the *MseI* Y (forked) adapter N containing the barcode. Restriction-ligation reactions were heat-inactivated at 65°C. The digested fragments with ligated adapters were purified with Agencourt AMPure XP (Beckman), and subjected to PCR amplification using Phusion Master Mix (NEB) with universal primer and index primer to add index and complete i5 and i7 sequences. The PCR products were purified using Agencourt AMPure XP paramagnetic beads (Beckman) and pooled. DNA quality was checked by running the samples on a 2% agarose gel. DNA fragments between 375 and 400 bp (with indexes and adaptors) in size were isolated using a Gel Extraction Kit (Qiagen). These fragments were then purified using Agencourt AMPure XP beads (Beckman) and diluted for sequencing. The paired-end sequencing was performed on the Illumina HiSeq 2000 platform (Illumina, USA). Raw GBS data have been deposited in the NCBI sequence read archive (SRA) under accession number PRJNA807126.

7.2.3 Sequence data analysis and SNP calling

Raw sequence data was assigned to each accession based on the sequences of the barcoded adapters. Three strict filtering steps were applied to obtain high-quality read data: (1) removal of paired reads with 10% or more unidentified nucleotides (N); (2) removal of paired reads with >50% bases having phred quality scores of <5; (3) removal of paired reads with barcode adapters. After quality control, the cleaned reads from each accession were aligned to the ‘Sanfensan’ reference genome using BWA

with the default settings. The results were converted to binary alignment map (BAM) format and sorted by reference position using SAMtools. Variant calling was performed using GATK. Only bi-allelic sites with MAF >0.05, missing data <20%, and heterozygous calls <10% were retained for downstream analyses. A total of 49,702 high-quality SNPs were obtained from the 659 oat lines.

7.2.4 Genome-wide association analysis

A genome-wide association analysis for the hulless trait was performed with TASSEL 5.0[78]. A mixed-linear model incorporating the kinship matrix and population structure (Q matrix) was used. The Q matrix was generated from the PCA results, while the K matrix was estimated by using the centered identity-by-state method implemented in TASSEL. Manhattan plots were generated using the "CMplot" R package. A genome-wide threshold of $-\log(P) = 6.70$, calculated from the formula "$-\log_{10}$(0.01/effective number of SNPs)" was used to identify marker loci that were associated with the hulless trait. A total of 32 markers were significantly associated with hulless trait. A strong peak on chromosome 4D was detected to be highly associated with the hulless grain trait.

7.2.5 Transcriptome comparison between hulled and hulless oats

Twelve diverse hulless and 10 hulled oat accessions were selected for RNA-seq analysis. The sequencing libraries were first constructed using an equal mixture of RNA from each of the seven RNA sample types described above. To detect the DEGs between hulled and hulless oats in the different tissues and developmental stages, another seven sequencing libraries were constructed for 'Sanfensan' (hulless oat) and 'Ogle' (hulled oat) using the seven RNA sample types isolated from each independently. RNA-seq was performed on an MGISEQ-2000 instrument at the

Genome Centre of GrandOmics (Wuhan, China). The amount of raw data obtained for each accession is given in **Supplementary Table 1**.

The DEGs between hulled and hulless oats as well as between different tissues/developmental stages were identified using the same methods as described in section 6.3.

7.2.6 Sequence comparison of gene *A.satnudSFS4D01G000045*

PCR was performed to amplify the genomic sequences of the candidate gene *A.satnudSFS4D01G000045* in five hulled (accession number or cultivar name: 'AC Aylmer', 'Ogle', 'Edmund', ZY001265, and ZY001332) and five hulless oat lines (accession number or cultivar name: 'Sanfensan', 'Baiyan_2', 'Baiyan_8', ZY001524, and ZY001693) (Supplementary Table 22) using a gene-specific primer pair: forward primer 5'-CCACAGATTTGGAGAGGAGATTT -3'; reverse primer: 5'-AAAGCAAAAAGTGGTAGACCTGA-3'. PCR amplification used the following protocol: an initial denaturation step at 95℃ for 5 min, followed by 35 cycles of 95℃ for 2 min, a 72℃ elongation for 1 min, and a final elongation step at 72℃ for 10 min. PCR reactions were performed with Platinum™ Taq DNA Polymerase (Thermo Fisher, USA) in 25 μl volumes following the manufacturer's recommendations. PCR products were purified using Agencourt AMPure XP beads (Beckman), cloned into the pGEM-T vector, and transformed into JM109 competent cells (Qingke, Beijing, China). We selected 8-10 positive clones from each sample and sequenced the inserts on an Applied Biosystems ABI DNA Analyzer (Sangon, Shanghai, China). Multiple sequence alignment was performed using the ClustalW (v2.0) program.

7.2.7 Kompetitive Allele-Specific PCR (KASP) validation

To test the correlation between the *A.satnudSFS4D01G000045* gene and the hulless

trait, a KASP marker was developed from the genomic sequence of the gene harboring a SNP that causes an amino acid change following standard KASP guidelines (https://www.lgcgroup.com/LGCGroup/media/PDFs/Products/Genotyping/KASP-genotyping-chemistry-User-guide.pdf) and tested in the diverse oat collection. PCR reaction mixtures (10 μL) containing 5 μl of 1× KASP master mixture, 2μl (20 ng/μl) of genomic DNA, 2 μl ddH_2O and 1 μl of the primer mixture (consisting of 15 μl reverse primer (10μm/ul), 6 μl of each forward primer (10μm/ul), and 23 μl ddH_2O). The KASP primer sequences are as follows:

Forward_1: 5'-AGCCGCTCTCGGAGCTGATCC-3';

Forward_2: 5'-AGCCGCTCTCGGAGCTGATCT-3';

Reverse: 5'-CCAGTTCACTCGCCTTCCACAGATT-3'

Amplification was carried out starting with a single cycle of 95°C for 15 min, followed by 10 touchdown cycles (95°C for 20 s; touchdown at 61°C initially and decreasing by 0.6°C per cycle for 1 min) and 25-35 cycles of annealing (95°C for 20 s; 55°C for 1 min). The whole process was performed on a real-time PCR system (Bio-Rad CFX96). End point genotyping was done using CFX Manager 3.1 software.

Supplementary References

1 Yan, H. et al. High-density marker profiling confirms ancestral genomes of *Avena* species and identifies D-genome chromosomes of hexaploid oat. *Theor. Appl. Genet.* **129**, 2133-2149, doi:10.1007/s00122-016-2762-7 (2016).

2 Zhuang, W. et al. The genome of cultivated peanut provides insight into legume karyotypes, polyploid evolution and crop domestication. *Nat. Genet.* **51,** 865-876, doi:10.1038/s41588-019-0402-2 (2019)

3 Marçais, G. & Kingsford, C. A fast, lock-free approach for efficient parallel counting of occurrences of k-mers. *Bioinformatics* **27**, 764-770, doi:10.1093/bioinformatics/btr011 (2011).

4 Wang, K. et al. African lungfish genome sheds light on the vertebrate water-to-land transition. *Cell* **184**, 1362-1376.e1318, doi:10.1016/j.cell.2021.01.047 (2021).

5 Yan, Q. et al. The elephant grass (*Cenchrus purpureus*) genome provides insights into anthocyanidin accumulation and fast growth. *Mol. Ecol. Resour.* **21**, 526-542, doi:10.1111/1755-0998.13271 (2021).

6 Yan, B. et al. A chromosome-level genome assembly of *Ephestia elutella* (Hübner, 1796) (Lepidoptera: Pyralidae). *Genome Biol. Evol.* **13**, evab114, doi:10.1093/gbe/evab114 (2021).

7 Bolger, A. M., Lohse, M. & Usadel, B. Trimmomatic: a flexible trimmer for Illumina sequence data. *Bioinformatics* **30**, 2114-2120, doi:10.1093/bioinformatics/btu170 (2014).

8 Li, H. Minimap2: pairwise alignment for nucleotide sequences. *Bioinformatics* **34**, 3094-3100, doi:10.1093/bioinformatics/bty191 (2018).

9 Vaser, R., Sovic, I., Nagarajan, N. & Sikic, M. Fast and accurate de novo genome assembly from long uncorrected reads. *Genome Res.* **27**, 737-746, doi:10.1101/gr.214270.116 (2017).

10 Maughan, P. J. et al. Genomic insights from the first chromosome-scale assemblies of oat (*Avena* spp.) diploid species. *BMC Biol.* **17**, 92,

doi:10.1186/s12915-019-0712-y (2019).

11 Alonge, M. et al. RaGOO: fast and accurate reference-guided scaffolding of draft genomes. *Genome Biol.* **20**, 224, doi:10.1186/s13059-019-1829-6 (2019).

12 Chen, S., Zhou, Y., Chen, Y. & Gu, J. fastp: an ultra-fast all-in-one FASTQ preprocessor. *Bioinformatics* **34**, i884-i890, doi:10.1093/bioinformatics/bty560 (2018).

13 Langmead, B. & Salzberg, S. L. Fast gapped-read alignment with Bowtie 2. *Nat. Methods* **9**, 357-359, doi:10.1038/nmeth.1923 (2012).

14 Burton, J. N. et al. Chromosome-scale scaffolding of de novo genome assemblies based on chromatin interactions. *Nat. Biotechnol.* **31**, 1119-1125, doi:10.1038/nbt.2727 (2013).

15 Li, H. & Durbin, R. Fast and accurate long-read alignment with Burrows-Wheeler transform. *Bioinformatics* **26**, 589-595, doi:10.1093/bioinformatics/btp698 (2010).

16 Li, H. et al. The Sequence Alignment/Map format and SAMtools. *Bioinformatics* **25**, 2078-2079, doi:10.1093/bioinformatics/btp352 (2009).

17 Mckenna, A. et al. The Genome Analysis Toolkit: A MapReduce framework for analyzing next-generation DNA sequencing data. *Genome Res.* **20**, 1297-1303, doi:10.1101/gr.107524.110 (2010).

18 Manni, M., Berkeley, M. R., Seppey, M., Simão, F. A. & Zdobnov, E. M. BUSCO Update: novel and streamlined workflows along with broader and deeper phylogenetic coverage for scoring of Eukaryotic, Prokaryotic, and Viral genomes. *Mol. Biol. Evol.* **38**, 4647-4654, doi:10.1093/molbev/msab199 (2021).

19 Ou, S., Chen, J. & Jiang, N. Assessing genome assembly quality using the LTR Assembly Index (LAI). *Nucleic Acids Res.* **46**, e126-e126, doi:10.1093/nar/gky730 (2018).

20 Ou, S. & Jiang, N. LTR_retriever: A highly accurate and sensitive program for

identification of long terminal repeat retrotransposons. *Plant Physiol.* **176**, 1410-1422, doi:10.1104/pp.17.01310 (2018).

21 Bekele, W. A., Wight, C. P., Chao, S., Howarth, C. J. & Tinker, N. A. Haplotype-based genotyping-by-sequencing in oat genome research. *Plant Biotechnol. J.* **16**, 1452-1463, doi:10.1111/pbi.12888 (2018).

22 Aragón-Alcaide, L., Miller, T., Schwarzacher, T., Reader, S. & Moore, G. A cereal centromeric sequence. *Chromosoma* **105**, 261-268, doi:10.1007/BF02524643 (1996).

23 Tomás, D. et al. Use of repetitive sequences for molecular and cytogenetic characterization of *Avena* species from Portugal. *Int. J. Mol. Sci.* **17**, 203, doi:10.3390/ijms17020203 (2016).

24 Jiang, W. et al. A universal karyotypic system for hexaploid and diploid *Avena* species brings oat cytogenetics into the genomics era. *BMC Plant Biol.* **21**, 213, doi:10.1186/s12870-021-02999-3 (2021).

25 Wu, T. D. & Watanabe, C. K. GMAP: a genomic mapping and alignment program for mRNA and EST sequences. *Bioinformatics* **21**, 1859-1875, doi:10.1093/bioinformatics/bti310 (2005).

26 Tang, S., Lomsadze, A. & Borodovsky, M. Identification of protein coding regions in RNA transcripts. *Nucleic Acids Res.* **43**, e78, doi:10.1093/nar/gkv227 (2015).

27 Keilwagen, J. et al. Using intron position conservation for homology-based gene prediction. *Nucleic Acids Res.* **44**, e89-e89, doi:10.1093/nar/gkw092 (2016).

28 Stanke, M., Diekhans, M., Baertsch, R. & Haussler, D. Using native and syntenically mapped cDNA alignments to improve de novo gene finding. *Bioinformatics* **24**, 637-644, doi:10.1093/bioinformatics/btn013 (2008).

29 Lomsadze, A., Burns, P. D. & Borodovsky, M. Integration of mapped RNA-Seq reads into automatic training of eukaryotic gene finding algorithm.

Nucleic Acids Res. **42**, e119-e119, doi:10.1093/nar/gku557 (2014).

30 Haas, B. J. et al. Automated eukaryotic gene structure annotation using EVidenceModeler and the program to assemble spliced alignments. *Genome Biol.* **9**, R7, doi:10.1186/gb-2008-9-1-r7 (2008).

31 Urasaki, N. et al. Draft genome sequence of bitter gourd (*Momordica charantia*), a vegetable and medicinal plant in tropical and subtropical regions. *DNA Res.* **24**, 51-58, doi:10.1093/dnares/dsw047 (2017).

32 Jones, P. et al. InterProScan 5: genome-scale protein function classification. *Bioinformatics* **30**, 1236-1240, doi:10.1093/bioinformatics/btu031 (2014).

33 Nawrocki, E. P. & Eddy, S. R. Infernal 1.1: 100-fold faster RNA homology searches. *Bioinformatics* **29**, 2933-2935, doi:10.1093/bioinformatics/btt509 (2013).

34 Griffiths-Jones, S. et al. Rfam: annotating non-coding RNAs in complete genomes. *Nucleic Acids Res.* **33**, D121-D124, doi:10.1093/nar/gki081 (2005).

35 Lagesen, K. et al. RNAmmer: consistent and rapid annotation of ribosomal RNA genes. *Nucleic Acids Res.* **35**, 3100-3108, doi:10.1093/nar/gkm160 (2007).

36 Lowe, T. M. & Eddy, S. R. tRNAscan-SE: a program for improved detection of transfer RNA genes in genomic sequence. *Nucleic Acids Res.* **25**, 955-964, doi:10.1093/nar/25.5.955 (1997).

37 Wang, X. & Wang, L. GMATA: an integrated software package for genome-scale SSR mining, marker development and viewing. *Front. Plant Sci.* **7**, doi:10.3389/fpls.2016.01350 (2016).

38 Benson, G. Tandem repeats finder: a program to analyze DNA sequences. *Nucleic Acids Res.* **27**, 573-580, doi:10.1093/nar/27.2.573 (1999).

39 Han, Y. & Wessler, S. R. MITE-Hunter: a program for discovering miniature inverted-repeat transposable elements from genomic sequences. *Nucleic Acids Res.* **38**, e199-e199, doi:10.1093/nar/gkq862 (2010).

40 Xu, Z. & Wang, H. LTR_FINDER: an efficient tool for the prediction of full-length LTR retrotransposons. *Nucleic Acids Res.* **35**, W265-W268, doi:10.1093/nar/gkm286 (2007).

41 Ellinghaus, D., Kurtz, S. & Willhoeft, U. LTRharvest, an efficient and flexible software for de novo detection of LTR retrotransposons. *BMC Bioinformatics* **9**, 18, doi:10.1186/1471-2105-9-18 (2008).

42 Bedell, J. A., Korf, I. & Gish, W. MaskerAid: a performance enhancement to RepeatMasker. *Bioinformatics* **16**, 1040-1041, doi:10.1093/bioinformatics/16.11.1040 (2000).

43 Flynn, J. M. et al. RepeatModeler2 for automated genomic discovery of transposable element families. *Proc. Natl. Acad. Sci. USA* **117**, 9451-9457, doi:10.1073/pnas.1921046117 (2020).

44 Abrusán, G., Grundmann, N., DeMester, L. & Makalowski, W. TEclass-a tool for automated classification of unknown eukaryotic transposable elements. *Bioinformatics* **25**, 1329-1330, doi:10.1093/bioinformatics/btp084 (2009).

45 Jurka, J. et al. Repbase Update, a database of eukaryotic repetitive elements. *Cytogenet. Genome Res.* **110**, 462-467, doi:10.1159/000084979 (2005).

46 Zhang, Z. et al. PseudoPipe: an automated pseudogene identification pipeline. *Bioinformatics* **22**, 1437-1439, doi:10.1093/bioinformatics/btl116 (2006).

47 Ranwez, V., Douzery, E. J. P., Cambon, C., Chantret, N. & Delsuc, F. MACSE v2: toolkit for the alignment of coding sequences accounting for frameshifts and stop codons. *Mol. Biol. Evol.* **35**, 2582-2584, doi:10.1093/molbev/msy159 (2018).

48 Pedersen, B. S. & Quinlan, A. R. Mosdepth: quick coverage calculation for genomes and exomes. *Bioinformatics* **34**, 867-868, doi:10.1093/bioinformatics/btx699 (2018).

49 Linares, C., Ferrer, E. & Fominaya, A. Discrimination of the closely related A and D genomes of the hexaploid oat *Avena sativa* L. *Proc. Natl. Acad. Sci.*

USA **95**, 12450, doi:10.1073/pnas.95.21.12450 (1998).

50 Fominaya, A., Hueros, G., Loarce, Y. & Ferrer, E. Chromosomal distribution of a repeated DNA sequence from C-genome heterochromatin and the identification of a new ribosomal DNA locus in the *Avena* genus. *Genome* **38**, 548-557, doi:10.1139/g95-071 (1995).

51 Wang, Y. *et al.* MCScanX: a toolkit for detection and evolutionary analysis of gene synteny and collinearity. *Nucleic Acids Res.* **40**, e49, doi:10.1093/nar/gkr1293 (2012).

52 Emms, D. M. & Kelly, S. OrthoFinder: phylogenetic orthology inference for comparative genomics. *Genome Biol.* **20**, 238, doi:10.1186/s13059-019-1832-y (2019).

53 Edgar, R. C. MUSCLE: multiple sequence alignment with high accuracy and high throughput. *Nucleic Acids Res.* **32**, 1792-1797, doi:10.1093/nar/gkh340 (2004).

54 Talavera, G. & Castresana, J. Improvement of phylogenies after removing divergent and ambiguously aligned blocks from protein sequence alignments. *Syst. Biol.* **56**, 564-577, doi:10.1080/10635150701472164 (2007).

55 Deng, C. & Wang Y. B. Oat-genome-origin-and-evolution: Oat genome origin and evolution (V1.0). *Zenodo* https;//doi.org/10.5281/zenodo.6622160 (2022).

56 Stamatakis, A. RAxML version 8: a tool for phylogenetic analysis and post-analysis of large phylogenies. *Bioinformatics* **30**, 1312-1313, doi:10.1093/bioinformatics/btu033 (2014).

57 Yang, Z. PAML 4: phylogenetic analysis by maximum likelihood. *Mol. Biol. Evol.* **24**, 1586-1591, doi:10.1093/molbev/msm088 (2007).

58 De Bie, T., Cristianini, N., Demuth, J. P. & Hahn, M. W. CAFE: a computational tool for the study of gene family evolution. *Bioinformatics* **22**, 1269-1271, doi:10.1093/bioinformatics/btl097 (2006).

59 Murat, F., Armero, A., Pont, C., Klopp, C. & Salse, J. Reconstructing the

genome of the most recent common ancestor of flowering plants. *Nat. Genet.* **49**, 490-496, doi:10.1038/ng.3813 (2017).

60 Grabherr, M. G. et al. Full-length transcriptome assembly from RNA-Seq data without a reference genome. *Nat. Biotechnol.* **29**, 644-652, doi:10.1038/nbt.1883 (2011).

61 Krzywinski, M. et al. Circos: an information aesthetic for comparative genomics. *Genome Res.* **19**, 1639-1645, doi:10.1101/gr.092759.109 (2009).

62 Schield, D. R. *et al.* The origins and evolution of chromosomes, dosage compensation, and mechanisms underlying venom regulation in snakes. *Genome Res.* **29**, 590-601, doi:10.1101/gr.240952.118 (2019).

63 Yan, H. et al. New evidence confirming the CD genomic constitutions of the tetraploid *Avena* species in the section *Pachycarpa* Baum. *PloS One* **16**, e0240703, doi:10.1371/journal.pone.0240703 (2021).

64 Fu, S. et al. Oligonucleotide probes for ND-FISH analysis to identify rye and wheat chromosomes. *Sci. Rep.* **5**, 10552, doi:10.1038/srep10552 (2015).

65 Rieseberg, L. H. Chromosomal rearrangements and speciation. *Trends Ecol. Evol.* **16**, 351-358, doi:10.1016/S0169-5347(01)02187-5 (2001).

66 Wooten, D. R. et al. An intergenomic reciprocal translocation associated with oat winter hardiness component traits. *Crop Sci.* **47**, 1832-1840, doi:10.2135/cropsci2006.12.0768 (2007).

67 Yan, H. et al. Genome size variation in the genus *Avena*. *Genome* **59**, 209-220, doi:10.1139/gen-2015-0132 (2016).

68 Suyama, M., Torrents, D. & Bork, P. PAL2NAL: robust conversion of protein sequence alignments into the corresponding codon alignments. *Nucleic Acids Res.* **34**, W609-612, doi:10.1093/nar/gkl315 (2006).

69 Kim, D., Langmead, B. & Salzberg, S. L. HISAT: a fast spliced aligner with low memory requirements. *Nat. methods* **12**, 357-360, doi:10.1038/nmeth.3317 (2015).

70 Anders, S., Pyl, P. T. & Huber, W. HTSeq--a Python framework to work with high-throughput sequencing data. *Bioinformatics* **31**, 166-169, doi:10.1093/bioinformatics/btu638 (2015).

71 Hollister, J. D. & Gaut, B. S. Epigenetic silencing of transposable elements: a trade-off between reduced transposition and deleterious effects on neighboring gene expression. *Genome Res.* **19**, 1419-1428, doi:10.1101/gr.091678.109 (2009).

72 Edger, P. P. et al. Origin and evolution of the octoploid strawberry genome. *Nat. Genet.* **51**, 541-547, doi:10.1038/s41588-019-0356-4 (2019).

73 Pertea, M. et al. StringTie enables improved reconstruction of a transcriptome from RNA-seq reads. *Nat. Biotechnol.* **33**, 290-295, doi:10.1038/nbt.3122 (2015).

74 Tardaguila, M. et al. SQANTI: extensive characterization of long-read transcript sequences for quality control in full-length transcriptome identification and quantification. *Genome Res.* **28**, 396-411, doi:10.1101/gr.222976.117 (2018).

75 Sanseverino, W. et al. PRGdb: a bioinformatics platform for plant resistance gene analysis. *Nucleic Acids Res.* **38**, D814-D821, doi:10.1093/nar/gkp978 (2010).

76 Simons, M. D. *The Cereal Rusts*. in *Diseases, Distribution, Epidemiology, and Control* (eds Alan P. Roelfs & William R. Bushnell) 131-172 (Academic Press, 1985).

77 Kebede, A. Z. et al. Mapping oat crown rust resistance gene *Pc45* confirms association with *PcKM*. *G3 (Bethesda)* **9**, 505-511, doi:10.1534/g3.118.200757 (2019).

78 Bradbury, P. J. et al. TASSEL: software for association mapping of complex traits in diverse samples. *Bioinformatics* **23**, 2633-2635, doi:10.1093/bioinformatics/btm308 (2007).
