# Supplementary material for: Reference genome assemblies reveal the origin and evolution of allohexaploid oat
Source: Nat Genet. 2022 Jul 18;54(8):1248–58. doi: 10.1038/s41588-022-01127-7 (PMC9355876; doi:10.1038/s41588-022-01127-7)
Supplement: Supplementary file 2 — Reporting Summary [file 41588_2022_1127_MOESM2_ESM.pdf]

nature portfolio

Corresponding author(s): Yuanying Peng, Tao Ma, Yuming Wei, Fei Lu & Changzhong Ren

Last updated by author(s): Jun 7, 2022

Reporting Summary

Nature Portfolio wishes to improve the reproducibility of the work that we publish. This form provides structure for consistency and transparency in reporting. For further information on Nature Portfolio policies, see our Editorial Policies and the Editorial Policy Checklist.

Statistics

For all statistical analyses, confirm that the following items are present in the figure legend, table legend, main text, or Methods section.

n/a	Confirmed	
☐	☒	The exact sample size (*n*) for each experimental group/condition, given as a discrete number and unit of measurement
☐	☒	A statement on whether measurements were taken from distinct samples or whether the same sample was measured repeatedly
☐	☒	The statistical test(s) used AND whether they are one- or two-sided<br>*Only common tests should be described solely by name; describe more complex techniques in the Methods section.*
☒	☐	A description of all covariates tested
☒	☐	A description of any assumptions or corrections, such as tests of normality and adjustment for multiple comparisons
☐	☒	A full description of the statistical parameters including central tendency (e.g. means) or other basic estimates (e.g. regression coefficient) AND variation (e.g. standard deviation) or associated estimates of uncertainty (e.g. confidence intervals)
☐	☒	For null hypothesis testing, the test statistic (e.g. *F*, *t*, *r*) with confidence intervals, effect sizes, degrees of freedom and *P* value noted<br>*Give P values as exact values whenever suitable.*
☒	☐	For Bayesian analysis, information on the choice of priors and Markov chain Monte Carlo settings
☒	☐	For hierarchical and complex designs, identification of the appropriate level for tests and full reporting of outcomes
☒	☐	Estimates of effect sizes (e.g. Cohen's *d*, Pearson's *r*), indicating how they were calculated

Our web collection on statistics for biologists contains articles on many of the points above.

Software and code

Policy information about availability of computer code

Data collection	No software was used for data collection
Data analysis	Guppy (v3.2.2), Jellyfish (v2.0), NextDenovo (v2.0-beta.1) (https://github.com/Nextomics/NextDenovo), NextCorrect, Trimmomatic (v0.40), mininmap2 (v2.18), Racon (v1.4.21), NextPolish (v1.0.5), RaGOO (v1.1), Bowtie2 (v2.3.2), fastp, LACHESIS (https://github.com/shendurelab/LACHESIS), BWA (v0.7.10-r789), SAMtools (v1.9), GATK (v4.1.9.0), BUSCO (v5.2.2), IsoSeq3(https://github.com/PacificBiosciences/IsoSeq), cDNA_Cupcake (v24.3.0) (https://github.com/Magdoll/cDNA_Cupcake), GMAP (release 2018-07-04), GeneMarkS-T (http://topaz.gatech.edu/GeneMark/license_download.cgi), GeMoMa (v1.6.1), AUGUSTUS (v2.4), BLASTn (v2.7.1), BLASTp (v2.7.1), GeneMark-ET (v4.0), EVidenceModeler (v1.1.1), TransposonPSI (v1.0.0), InterProScan (v5.22), Infernal (v1.1.2), RNAmmer (v1.2), tRNAscan-SE (v2.0), miRanda (v3.0), GMATA (v2.2), Tandem Repeats Finder (v4.07b), MITE-hunter (http://target.iplantcollaborative.org/mite_hunter.html), ClustalW, LTR_FINDER (v1.0.5), LTR_harvest (v1.5.10), LTR_retriever (v2.8), RepeatMasker (v1.331), RepeatModeler (v2.01), TEclass, Repbase (v19.06), Pseudopipe, MACSE (v2), R (v4.05), Mosdepth (v0.3.0), MCScanX (git-97e74f40), MUSCLE (v3.8.31), OrthoFinder (v2.2.7), Gblocks (v0.9b), RAxML (v.8.2.7), PAML (v4.7), FigTree (v1.4.0), CAFÉ (v5.2.1), Trinity (v2.0.3), TransDecoder (v5.5.0), NOVOPlasty (v3.7), Circos (v0.69-9), Photoshop (v7.0), PAL2NAL (v14), HTseq (v0.9.1), HISAT2 (v2.2.1), StringTie (v2.2.0), SQANTI3 (v5.0), DeepCoil (v2.0.1), edgeR (v3.38.1), TASSEL 5.0.<br>The custom codes used to generate the results reported in the study are available at Github (https://github.com/YuboWang1994/Oat-genome-origin-and-evolution/tree/V1.0) and were also archived on Zenodo with DOI: https://doi.org/10.5281/zenodo.6622160.

For manuscripts utilizing custom algorithms or software that are central to the research but not yet described in published literature, software must be made available to editors and reviewers. We strongly encourage code deposition in a community repository (e.g. GitHub). See the Nature Portfolio guidelines for submitting code & software for further information.

Data

Policy information about availability of data

All manuscripts must include a data availability statement. This statement should provide the following information, where applicable:

- Accession codes, unique identifiers, or web links for publicly available datasets
- A description of any restrictions on data availability
- For clinical datasets or third party data, please ensure that the statement adheres to our policy

The genome assemblies and sequence data for A. sativa ssp. nuda cv. 'Sanfensan', A. insularis (CN 108634) and A. longiglumis (CN 58139) were deposited at NCBI under BioProject codes PRJNA 727473, PRJNA731599 and PRJNA716144, respectively. 'Sanfensan' genome assembly (SAMN19770945), ONT data (SAMN19021785), Hi-C data (SAMN19340419), NGS data (SAMN19582572), Iso-seq data (SAMN19581880) and RNA-seq data (SAMN19582573, SAMN19582574); A.insularis genome assembly (SAMN19771048), ONT data (SAMN19291344), Hi-C data (SAMN19312172), NGS data (SAMN19579880) and Iso-seq data (SAMN19581879); A.longiglumis genome assembly (SAMN19771099), ONT data (SAMN18395928), NGS data (SAMN19523931) and Iso-seq data (SAMN19581877). The genotyping-by-sequecing data for 659 oat lines were deposited at NCBI under BioProject code PRJNA807126. All raw data for the other 14 deep-sequenced accessions including eight diploids, five tetraploids and one hexaploid are available under project numbers that can be found in Supplementary Table 1. Functional annotation of the genomes used the SwisspProt (ftp.uniprot.org/pub/databases/uniprot/current_release/knowledgebase/complete/uniprot_sprot.fast.gz), NR (ftp.ncbi.nlm.nih.gov/blast/db/FASTA/nr.gz), KEGG (release 97, https://www.genome.jp/kegg/kegg2.html), KOG (ftp://ftp.ncbi.nih.gov/pub/COG/KOG/kyva),GO (http://purl.obolibrary.org/obo/go/go-basic.obo) databases. Non-coding RNA annotation used the Rfam database (http://ftp.ebi.ac.uk/pub/databases/Rfam/14.2/Rfam.tar.gz). Repetitive element annotation used the Repbase databse (RepBase19.06.embl.tar.gz). The OT3098 v2 (https://wheat.pw.usda.gov/jb?data=/ggds/oat-ot3098v2-pepsico) and hexaploid bread wheat (https://urgi.versailles.inrae.fr/download /iwgsc/IWGSC_RefSeq_Assemblies/v1.1/) reference genomes were retieved from the GrainGenes database.

Field-specific reporting

Please select the one below that is the best fit for your research. If you are not sure, read the appropriate sections before making your selection.

☒ Life sciences ☐ Behavioural & social sciences ☐ Ecological, evolutionary & environmental sciences

For a reference copy of the document with all sections, see nature.com/documents/nr-reporting-summary-flat.pdf

Life sciences study design

All studies must disclose on these points even when the disclosure is negative.

Sample size	No statistical methods were required to establish sample size for this study. The Sanfensan cultivar was chosen as a one representative hulless oat since this cultivar has a long cultivated history in China. Tetraploid (A. insularis) and diploid (A. longiglumis) species were chosen as the likely ancestors of hexaploid based on previous marker study. Fourteen additional Avena taxa were used to elucidate the evolution of genus Avena. These Avena taxa represent all genome types found among the extant Avena species. A panel of 659 oat lines from 52 countries or districts were subjected to genotyping-by-sequencing and used subsequently for GWAS analysis. It samples all regions where oat is spread.
Data exclusions	No data were excluded from analysis. Raw sequencing data was quality filtered as described in manuscript.
Replication	Bootstrapping for phylogenetic analyses based singly copy genes from 23 subgenomes and chloroplast genomes were replicated 100 times, while bootstrapping for phylogenetic analyses based whole genome SNPs were replicated 200 times. Three biological replicates were executed for the RNA-seq and expression analysis. For FISH karyotyping, at least three slides for each accession and ten chromosomes per slide were examined. All attempts at replication were successful.
Randomization	Randomizations were not needed for this study, which involved analyzing subgenomes residing within a nucleus of a single genotype. Plants were grown in a sterile growth chamber.
Blinding	Group allocation was not relevant to this study, so blinding was not necessary.

Reporting for specific materials, systems and methods

We require information from authors about some types of materials, experimental systems and methods used in many studies. Here, indicate whether each material, system or method listed is relevant to your study. If you are not sure if a list item applies to your research, read the appropriate section before selecting a response.

Materials & experimental systems

n/a	Involved in the study
☒	☐ Antibodies
☒	☐ Eukaryotic cell lines
☒	☐ Palaeontology and archaeology
☒	☐ Animals and other organisms
☒	☐ Human research participants
☒	☐ Clinical data
☒	☐ Dual use research of concern

Methods

n/a	Involved in the study
☒	☐ ChIP-seq
☒	☐ Flow cytometry
☒	☐ MRI-based neuroimaging
