# Supplementary figures and images for: Reference genome assemblies reveal the origin and evolution of allohexaploid oat
Source: Nat Genet. 2022 Jul 18;54(8):1248–58. doi: 10.1038/s41588-022-01127-7 (PMC9355876; doi:10.1038/s41588-022-01127-7)

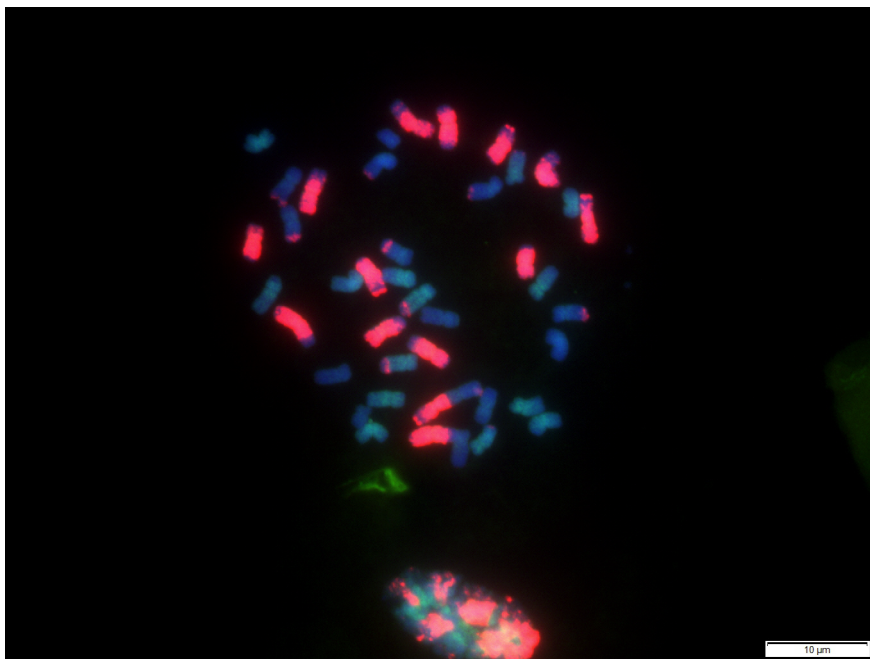

Fig 4c

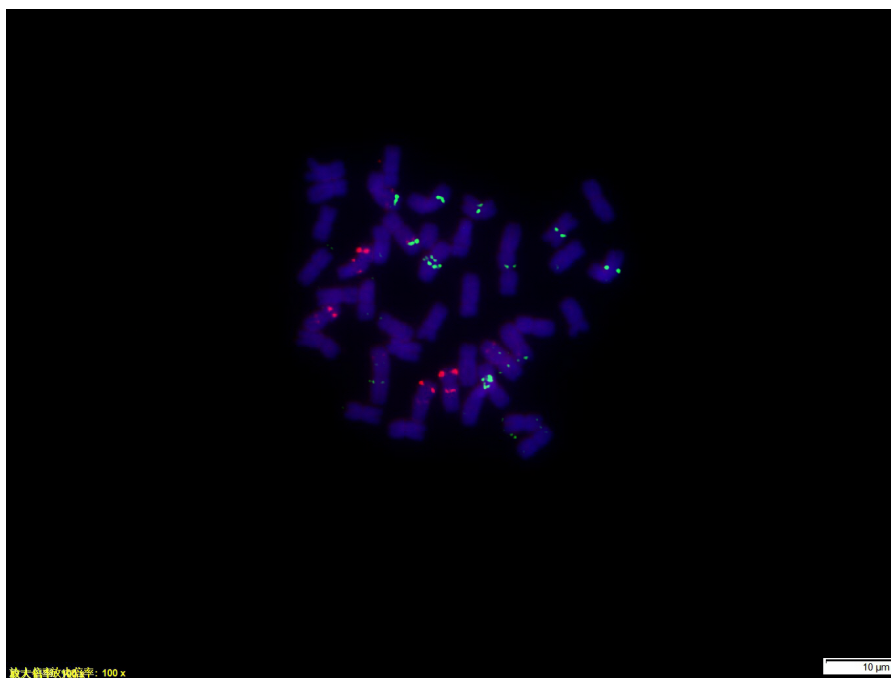

Fig 4d - sanfensan 3C

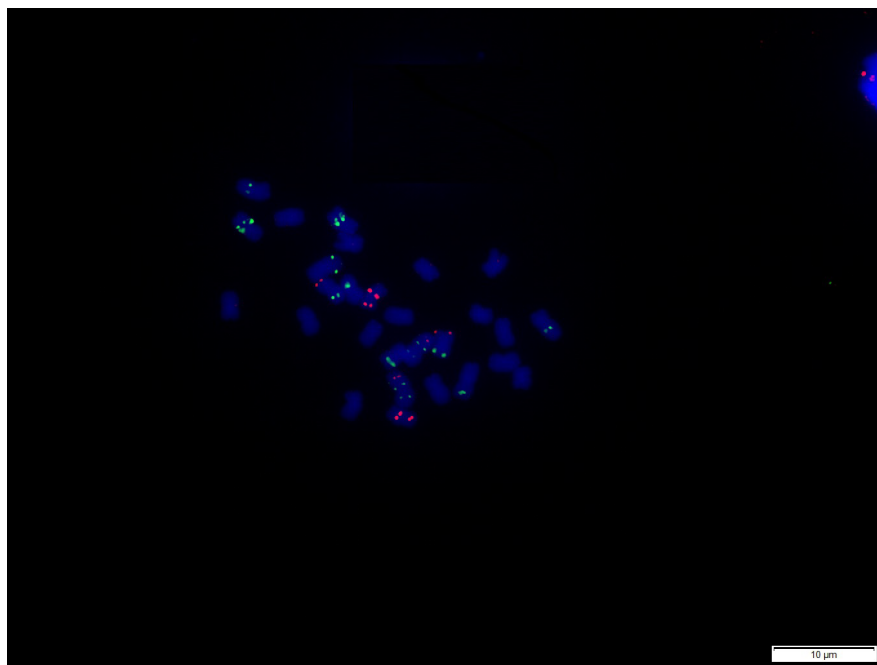

Fig 4d - *A. insularis* 3C

Supplement: Source Data Fig. 4 — Statistical Source Data and unprocessed FISH. [file 41588_2022_1127_MOESM9_ESM.pdf]
