# Supplementary material for: Reference genome assemblies reveal the origin and evolution of allohexaploid oat
Source: Nat Genet. 2022 Jul 18;54(8):1248–58. doi: 10.1038/s41588-022-01127-7 (PMC9355876; doi:10.1038/s41588-022-01127-7)
Supplement: Source Data Extended Data Fig. 6 — Statistical Source Data and unprocessed FISH. [file 41588_2022_1127_MOESM17_ESM.pdf]

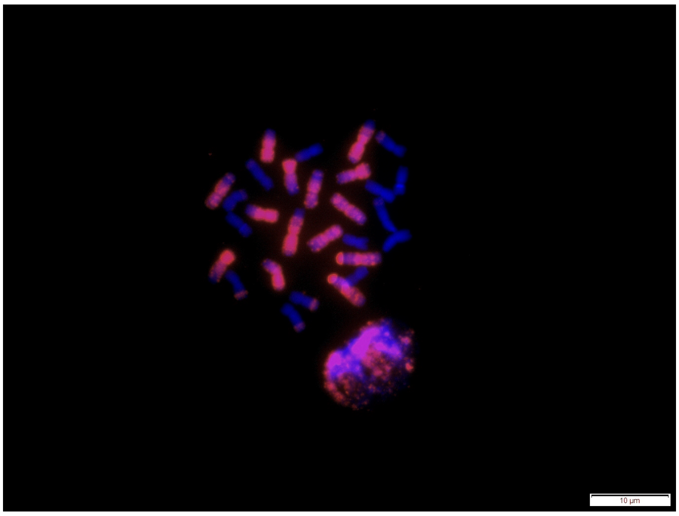

Extended Data Fig 6e

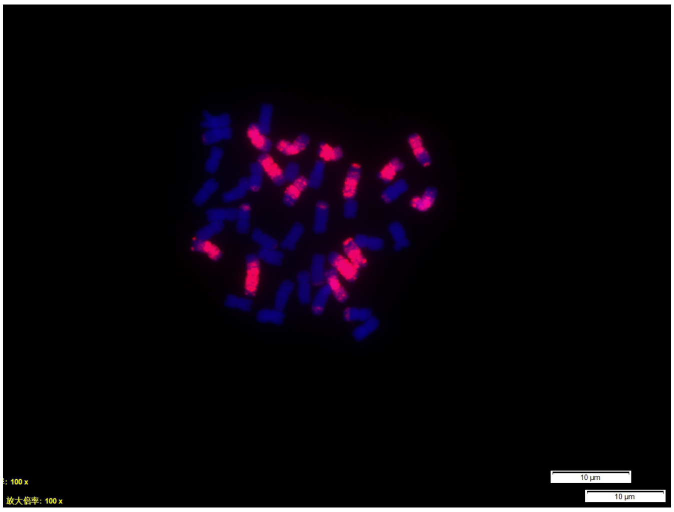

Extended Data Fig 6f sanfensan Am1

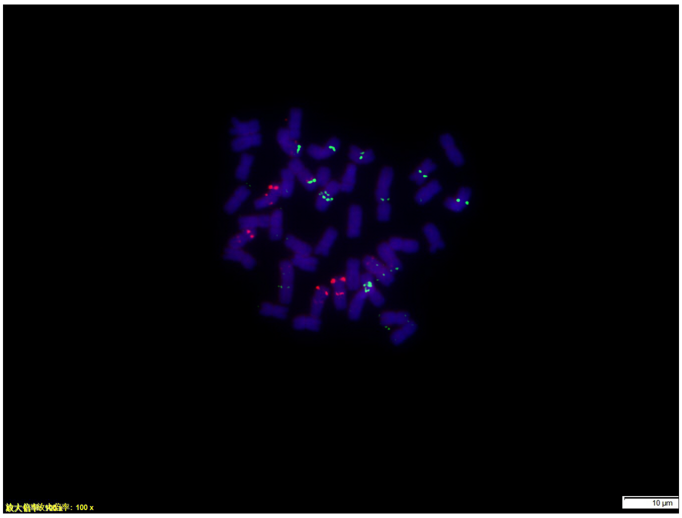

Extended Data Fig 6f sanfensan Oligo- 5SrDNA plus Oligo-6C343

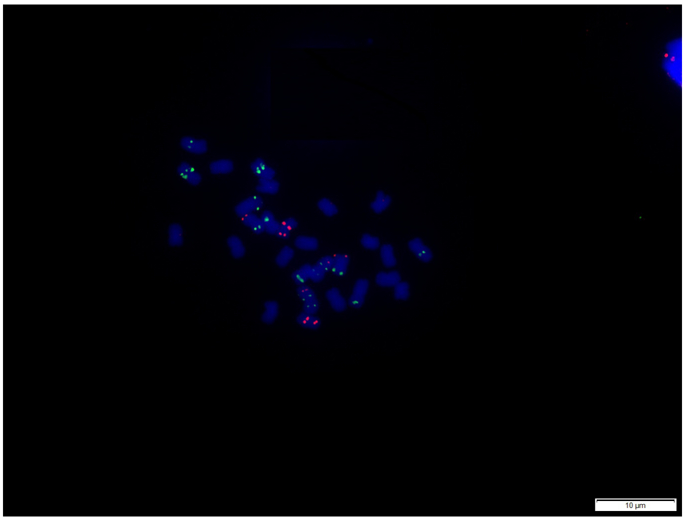

Extended Data Fig 6f *A. insularis* Oligo- 5SrDNA plus Oligo-6C343

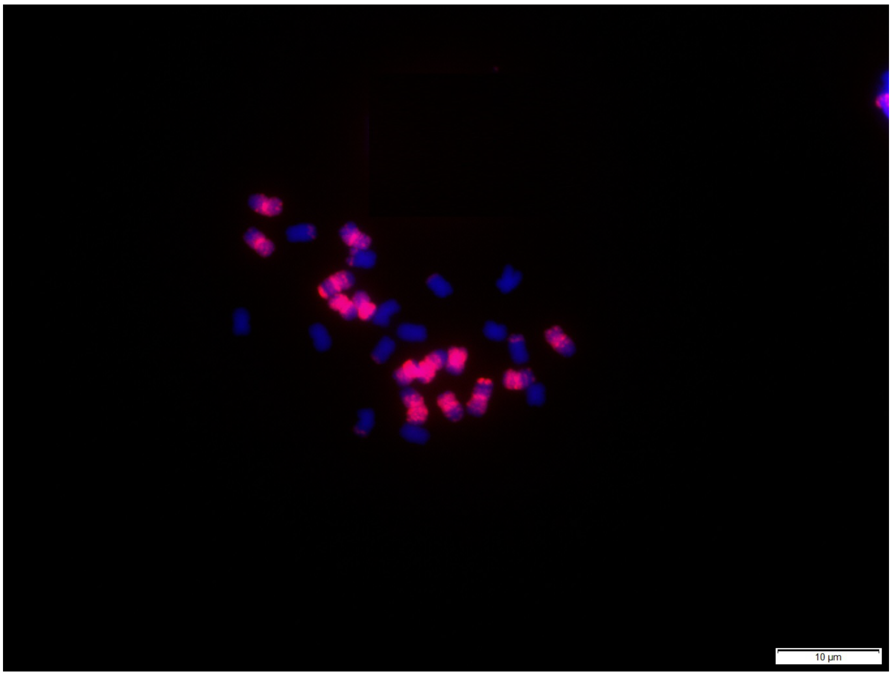

Extended Data Fig 6f *A. insularis* Am1
